# Sphingomyelin Phodiesterase Acid-Like 3A Promotes Hepatocellular Carcinoma Growth Through the Enhancer of Rudimentary Homolog

**DOI:** 10.3389/fonc.2022.852765

**Published:** 2022-05-24

**Authors:** Yu Zhang, Weipeng Chen, Xin Cheng, Feiran Wang, Cheng Gao, Fei Song, Fengliang Song, Xiaoliang Liang, Wanzhi Fang, Zhong Chen

**Affiliations:** ^1^Department of Hepatobiliary Surgery, Affiliated Hospital of Nantong University, Nantong, China; ^2^School of Medicine, Nantong University, Nantong, China; ^3^Department of General Surgery, Binhai County People’s Hospital, Yancheng, China; ^4^Department of General Surgery, Jingjiang People’s Hospital, Taizhou, China

**Keywords:** hepatocellular carcinoma (HCC), sphingomyelin phodiesterase acid-like 3A, enhancer of rudimentary homolog (ERH), cell migration, cell proliferation, apoptosis, prognosis

## Abstract

**Background:**

Hepatocellular carcinoma (HCC) is one of the most common malignant tumors worldwide, with unclear pathogenesis. Sphingomyelin phodiesterase acid-like 3A (SMPDL3A) affects cell differentiation and participates in immune regulation. However, its molecular biological function in HCC has not yet been elucidated.

**Methods:**

Data from 180 HCC patients were analyzed the relationship between the expression of SMPDL3A in liver cancer tissues and the prognosis of liver cancer patients. Crispr-Cas9 dual vector lentivirus was used to knock out SMPDL3A in HCC cell lines. The effects of SMPDL3A on cell viability were determined by CCK8 assay, clone formation experiment, cell cycle assay, cell scratch, TUNEL experiment and flow cytometry. Xenograft tumor assays in BALB/c nude mice confirmed that SMPDL3A promoted tumor growth and *in vivo*. Preliminary exploration of SMPDL3A interacting protein by mass spectrometry analysis and co-immunoprecipitation.

**Results:**

This study showed that the expression of SMPDL3A in HCC tissue differed from that in tumor-adjacent tissues. Moreover, the overall survival rate and tumor-free survival rate of patients with high-SMPDL3A expression were significantly lower than those with low-SMPDL3A expression. SMPDL3A expression was closely related to the level of protein induced by PIVKA-II, liver cirrhosis, tumor diameter, microvascular invasion, and Barcelona clinic liver cancer staging. Thus, SMPDL3A is an independent risk factor that affects the tumor-free survival rate and overall survival rate of HCC patients. *In vitro* study using Crispr-Cas9 genome editing technology revealed the knockout effect of *SMPDL3A* on cell proliferation, apoptosis, and migration. Cell counting kit-8 assay and clone formation experiment showed that sgSMPDL3A inhibited tumor cell proliferation and migration. Flow cytometry and TUNEL assay showed that sgSMPDL3A promoted apoptosis in tumors. Moreover, sgSMPDL3A inhibited tumor growth during subcutaneous tumor formation in nude mice. Immunohistochemistry of Ki67 and PNCA also indicated that sgSMPDL3A inhibited subcutaneous tumor proliferation in tumor-bearing nude mice. Further experiments showed that SMPDL3A interacts with the enhancer of rudimentary homolog (ERH).

**Conclusions:**

High-SMPDL3A expression was related to poor prognosis of patients with HCC. Knockout of *SMPDL3A* inhibited the proliferation and migration and accelerated the migration of HCC cells. SMPDL3A interacted with ERH to affect the tumorigenesis and progression of HCC.

## Introduction

As the most common type of primary liver cancer, hepatocellular carcinoma (HCC) ranks third among the causes of cancer-related deaths and sixth in the incidence worldwide (1). The World Health Organization predicts that the incidence of HCC will continue to rise in the future, and more than 1 million people are expected to die of HCC by 2030. China is a country with a high prevalence of liver diseases, with the incidence of HCC among male and female patients ranking first in the world (2, 3). Many studies have confirmed that HCC tumorigenesis is a complex process, involving multiple genes and multistep changes. The activation of related proto-oncogenes and the inactivation of tumor suppressor genes are the main causes of tumorigenesis and tumor progression (3). However, the specific molecular mechanism underlying tumorigenesis and tumor progression is not yet fully understood. Further exploration of new functional genes in HCC and their molecular mechanisms affecting tumorigenesis and tumor progression of HCC will provide important guidance for revealing the pathogenesis of HCC and for developing new therapeutic drugs to improve prognosis.

Our previous study has shown that the expression of voltage-dependent anion channel 1 (VDAC1) is closely related to the tumorigenesis and progression of HCC. MicroRNA-7 may bind to the 3′non-coding region of VDAC1 mRNA to promote tumor cell proliferation and invasion (4). Nevertheless, tumorigenesis and progression of HCC are complex processes, and the specific mechanism of VDAC1 affecting the proliferation and invasion of HCC has not yet been elucidated. To further study the role of downstream related molecules of VDAC1, we utilized the Affymetrix GeneChip system to analyze the downstream gene expression profile of *VDAC1* and showed 522 differentially expressed genes (DEGs) in HCC cells after knocking down *VDAC1*. Based on these findings, and combined with other literature, 17 representative DEGs were selected for further high-throughput functional screening based on cell proliferation. Knockdown of sphingomyelin phodiesterase acid-like 3A gene (*SMPDL3A*) showed a significant inhibitory effect on the proliferation of HCC cells. However, no relevant study on the mechanism of SMPDL3A in HCC was available in the current literature. Hence, we selected *SMPDL3A* for further investigation.

SMPDL3A has nucleotide phosphodiesterase activity and phosphoramidate activity, which may be related to the anabolism of the bisamidate prodrug in the liver (5). Studies have shown that SMPDL3A may affect cell differentiation and participate in immune regulation. Our latest research showed that SMPDL3A was expressed differently in HCC tissues and tumor-adjacent liver tissues. Through 180 pairs of tissue microarrays of patients with HCC, we showed that the SMPDL3A expression level was related to abnormal prothrombin, liver cirrhosis, microvascular invasion, and Barcelona clinic liver cancer (BCLC) staging. Patients with high-SMPDL3A expression had significantly lower tumor-free survival rate and overall survival rate than those with low-SMPDL3A expression. Moreover, knockout of *SMPDL3A* using Crispr-Cas9 genome editing technology in liver cancer cell lines significantly inhibited proliferation and migration and promoted apoptosis in cancer cells. In-depth study of the relationship between the expression of SMPDL3A and the tumorigenesis and progression of HCC showed that inhibition of SMPDL3A expression suppressed the proliferation of HCC and promoted tumor cell apoptosis. These results provide a novel target for in-depth understanding of the prevention and treatment of HCC.

## Materials and Methods

### Tissue Sampling and Clinical Data

HCC and tumor-adjacent tissues (> 2 cm) from patients who underwent radical surgical resection of HCC at the Department of Hepatobiliary Pancreatic Splenic Surgery, Affiliated Hospital of Nantong University, Jiangsu Province, China from January 2013 to June 2020 were collected. The fresh tissues were aliquoted and stored at 80°C for total protein and RNA extraction. The formalin-fixed tissues were stored at −4°C for tissue microarray. All histopathological sections were carefully diagnosed as HCC by two experienced pathologists. Patients’ data were retrieved from the information system of our hospital to record the length of hospitalization, age, sex, chronic hepatitis B infection, HCC tumor marker expression, Child–Pugh score, tumor number, tumor diameter, portal vein tumor thrombus, microvascular invasion, BCLC staging, and TNM staging. Patients with Class C Child–Pugh scores, positive surgical margins, preoperative intervention, radiotherapy and chemotherapy, targeted therapy and immunotherapy history, and incomplete clinical data were excluded. All included patients were followed up to record their postoperative recurrence and survival. Through data sorting, 180 pairs of HCC and tumor-adjacent tissues were used for immunohistochemistry. The clinical and follow-up data of the included patients with HCC patients were subjected to statistical analysis (**Table 1**). All samples were collected with the patient’s informed consent. The research protocol of this study was approved by the Ethics Committee of the Affiliated Hospital of Nantong University.

**Table 1:** Relationship between the expression of SMPDL3A in HCC tissues and clinical characteristics of HCC patients.

Clinical Valuable	Patient number (total=180)	SMPDL3A expression		P value
		High expression (n=107)	Low expression (n=73)	
Gender^a^				
Male	156	95	61	0.374
Female	24	12	12	
Age(years)^b^	180	107(59.69±9.92)	72(59.29±10.03)	0.940
AFP^a^				
Positive≥8,78ng/ml	102	58	49	0.447
Negative<8,78ng/m	78	44	29	
GGT-II^a^				
Positive	92	61	31	0.090
Negative	60	31	29	
PIVKA-II^a^				
Positive≥40 mAU/ml	100	68	32	0.013
Negative<40 mAU/ml	33	14	19	
HBsAg^a^				
Positive	137	79	58	0.477
Negative	43	28	15	
HBsAb^a^				
Positive	145	84	61	0.447
Negative	35	23	12	
HBeAg^a^				
Positive	82	43	39	0.094
Negative	98	64	34	
HBeAb^a^				
Positive	120	74	46	0.423
Negative	60	33	27	
HBcAb^a^				
Positive	172	102	70	1
Negative	8	5	3	
HBV-DNA^a^				
Positive ≥10^3^ /copies	71	45	26	0.439
Negative<10^3^ /copies	109	62	47	
Child-Pugh classification^a^				
Child-Pugh A	155	91	64	0.667
Child-Pugh B	25	16	9	
Tumor number^a^				
Single	153	94	59	0.209
Multi	27	13	14	
Tumor size[n(mean±SD)]^b^		107(5.81±3.49)	73(2.94±1.65)	<0.001
Cirrhosis^a^				
Present	104	54	50	0.021
Absent	76	53	23	
PVTT^a^				
Present	17	12	5	0.439
Absent	163	95	68	
MVI^a^				
Present	27	22	5	0.011
Absent	153	85	68	
Tumor differentiation^a^				
Poor	34	20	14	0.885
Moderate	140	84	56	
Well	6	3	3	
BCLC stage^a^				
Stage 0	33	9	24	<0.001
Stage A	87	45	42	
Stage B	45	43	2	
Stage C	15	10	5	
pTNM stage^a^				
I	144	85	59	0.122
II	18	8	10	
III	18	14	4	

MVI, microvascular invasion; PVTT, portal vein tumor thrombus, AFP; α-fetoprotein; pTNM, tumor, lymph node, metastasis classification.

P<0.05 was considered statistically significant.

a Student’s test.

b χ^2^ test.

### Cell Cultures

In this study, six HCC cell lines, including SNU-384, SNU-182, Sk-Hep-1, Hep3B, Huh7, and HepG2, were purchased from the Cell Bank of Type Culture Collection of Academy of Sciences. The highly invasive HCC cell line, HCC-LM3, and the human HCC cell line QGY7701 were gifts from a laboratory of the Eastern Hepatobiliary Surgery Hospital affiliated to the Naval Military Medical University, Shanghai, China. The HCC-LM3, HepG2, and Huh7 cells were incubated in Dulbecco’s modified eagle medium containing 10% fetal bovine serum. The QGY770, SNU-384, and SNU-182 cells were incubated in RPMI-1640 medium supplemented with 10% fetal bovine serum. The Hep3B and SK-Hep-1 HCC cell lines were incubated in MEM supplemented with 10% fetal bovine serum, GlutaMAX, non-essential amino acids, and 100 mM sodium pyruvate solution. All cells were cultured in a humidified incubator at 37°C and 5% CO_2_. The fetal bovine serum, culture medium, and reagents were purchased from Invitrogen (Waltham, MA).

### Affymetrix Whole-Genome Expression Profiling

After collecting the total RNA for analysis in an Agilent 2100 Bioanalyzer, the amplified RNA (aRNA) was prepared using the GeneChip 3′IVT Express Kit (Thermo Fisher Scientific (Waltham, MA), i.e., obtaining cDNA through one-strand synthesis, followed by obtaining double-stranded DNA templates and biotin-labeled aRNA through two-strand synthesis and *in vitro* inversion, respectively. The aRNA was purified, fragmented, and hybridized with the chip probe. After the hybridization was completed, the chip was washed and stained before photographing and obtaining the original data by scanning.

### Fluorescence-Based Real-Time Quantitative PCR (RT-qPCR)

After extracting the total RNA using TRIzol Reagent (Invitrogen), the total RNA was reverse transcribed into cDNA using Takara RT-PCR Master Mix (DRR0036A). The RT-qPCR system, LightCycler 480 Instrument II and SYBR Green solution (Takara) were used to detect the relative expression of the target gene, *SMPDL3A* using *β-actin* as an internal reference. The primers used in this study were synthesized by Sangon Biotech (Shanghai) Co., Ltd., China, with the following primer sequences: *SMPDL3A* forward primer: 5′-CTCACAGAGACAGCATTATGGTT-3′; *SMPDL3A* reverse primer: 5′-TTCACTGGTGTAACAGCAGGA-3′; β-actin forward primer: 5′-GGCGGCACCACCATGTACCCT-3′; β-actin reverse primer: 5′-AGGGGCCGGACTCGTCATACT-3′.

### High-Throughput Functional Screening Based on Cell Proliferation and Analysis of Cell Growth Curves

The experimental methods for high-throughput functional screening based on cell proliferation and specific interference sequences used in this study have been described previously (6). The Huh7 cells were infected with knockdown (KD) or healthy control (NC) lentivirus and inoculated into 96-well plates. A fluorescence microscope was used to observe the green florescence protein (GFP) expression. The cells at 80% confluence were harvested for further experiments. A cytology array-scanning system was used to analyze 200 cells per well daily. The cells were quantified by adjusting the input parameters based on the green-fluorescent signal measured in each well. The collected data were subjected to statistical analysis using a 5-day cell proliferation curve. The cell counts on the scanned images were measured using image analysis software. The number of cells at each time point was compared with the number of cells on the first day to obtain the cell proliferation rate of each group at each time point. The cell growth curve was prepared using the change in the multiplication factor. The cell proliferation rate was calculated using the following equation: Fold change (normal control group *vs.* knockdown group) = cell proliferation rate on day 5 in the normal control group/cell proliferation rate on day 5 in the knockdown group. A ≥ 2-fold change in cell proliferation rate indicated that cell proliferation was sufficiently slowed down. The effect of RNA interference (RNAi) lentivirus infection on cell proliferation was then measured.

### Immunohistochemistry and Semiquantitative Analysis

Diluted SMPDL3A polyclonal antibodies (1:1,000, Thermo Fisher Scientific) were dropped evenly on a tissue chip containing 180 pairs of HCC tissues and tumor-adjacent liver tissue, followed by incubating the chip overnight in a moisture chamber at 4°C. The corresponding secondary antibodies (Thermo Fisher Scientific) at a 1:1,000 dilution was evenly added to the tissue chip, which was further incubated at room temperature for 30 min. Three representative images were captured from each sample using a microscope and were subjected to double-blind analysis by a senior pathologist. Immunohistochemistry of SMDPL3A was scored from 0 to 12, and the percentage of positively stained tumor cells and the staining intensity of SMDPL3A immunohistochemistry were used for semiquantitative analysis of SMDPL3A expression as follows: score 0 refers to no staining; score 1 refers to light staining; score 2 refers to moderate staining; and score 3 refers to heavy staining. Analysis based on the percentage of positively stained cells was performed as follows: score 0 refers to < 5% positive staining; score 1 refers to 5%–25% positive staining; score 2 refers to 26%–50% positive staining; score 3 refers to 51%–75% positive staining; and score 4 refers to > 75% positive staining. Multiplication of two scores obtained based on the staining intensity and percentage of positively stained cells was used for semiquantitative analysis. A total score > 6 was defined as tissue with high-SMDPL3A expression, and a total score ≤ 6 was defined as tissue with low-SMDPL3A expression.

### Knocking Out *SMPDL3A* Using a Crispr-Cas9 Lentiviral Vector

The HCC cell lines Huh7 and HepG2 in the logarithmic growth phase were inoculated into 6-well plates (5 × 10^4^ cells per well) and incubated for 1 day. When the HCC cells had reached 50% confluence, they were transfected with the lentiviral vector Lenti-CAS9 (purchased from Shanghai Genechem Co., Ltd., Shanghai, China). Puromycin screening was performed 3 days after transfection to select the cancer cells to further transfer with single guide-SMPDL3A RNA (sgSMPDL3A, Shanghai Genechem Co., Ltd.). The GFP expression was observed under a fluorescence microscope. The HCC cells were harvested for analysis or related functional studies once they reached > 80% confluence.

### Detection of SgSMPDL3A Activity by the Surveyor Assay

Seven days after lentiviral transfection, the liver cells were harvested to extract the hybrid clone genome, followed by PCR amplification and annealing to obtain hybrid DNA products, which were cooled naturally to < 40°C. An enzyme digestion screening system was prepared in sterile PCR tubes to screen sgSMPDL3A. After incubating at 45°C for 20 min, the stopping solution was added to terminate the reaction, and the product was detected by 2% agarose-gel electrophoresis. Compared to the control group, a cleaved DNA band at the expected position was observed in the experimental group, indicating that sgSMPDL3A was active and ready for use in the subsequent experiments.

### Clonogenic Assay

The HepG2 and Huh7 cells of the experimental and control groups in the logarithmic growth phase were inoculated into 6-well plates (1,000 cells per well) for 30-day culture and colony formation. At the specified time point, 4% paraformaldehyde was added to fix the cells, followed by Giemsa staining for 10 min and several washes before counting the number of colonies under a light microscope(DM IL LED, Leica).

### Cell Counting Kit-8 (CCK-8)

The HepG2 and Huh7 cells in the logarithmic growth phase were inoculated into a 96-well plate (5,000 cells per well) and cultured for approximately 1 day until they had adhered to the walls of the wells before the viability assay. The CCK-8 reagent (Dojindo Molecular Technologies, Inc., Japan) was added to the cells at 24, 48, 72, and 96 h, followed by an hour of incubation and subsequent detection of the absorbance at 450 nm wavelength using a microplate reader.

### Detection of Apoptosis

Detection of apoptosis in the HCC cells was performed using TUNEL staining and APC Annexin V flow cytometric analysis. For TUNEL staining, the TUNEL staining kit (C1090, purchased from Beyotime Biotechnology, Shanghai, China) was used to stain the HCC cells according to the manufacturer’s instructions. The TUNEL-positively stained cells were observed under an upright fluorescence microscope(DM IL LED, Leica). In APC Annexin V flow cytometric analysis, HCC cell suspensions of the control and experimental groups were washed with binding buffer, followed by adding 10 µl APC-Annexin V dye for staining in the dark for 10 to 15 min before detection by a flow cytometer (Guava easyCyte HT, Millipore).

### *In Vitro* Scratch Assay

The HepG2 and Huh7 HCC cells in the logarithmic growth phase were inoculated into 6-well plates, followed by using a 20-µl pipette tip to create a vertical scratch once the cells had reached confluence. After washing the cells with phosphate buffered saline (PBS) to remove the scratched cells, the cancer cells were cultured in serum-free medium, followed by photographing at 0, 24, 48, and 72 h under a light microscope (DM IL LED, Leica). ImageJ software was used to calculate the average value of the distances between cells.

### Subcutaneous Tumor Formation in Nude Mice

To generate the HCC cell inoculation mouse model, 4-week-old BALB/c nude mice (20 females, body weight of 18-21g) were purchased from the Animal Research Center of Nantong University. The mice were housed in a specific barrier environment (21°C, 60% humidity) on a 12h-12h light-dark cycle. All experiments were conducted under the guidance of the care and use of laboratory animals issued by the Ministry of Science and Technology of the China. After one-week adaptive feeding, the nude mice were randomly divided into two groups (n=10 per group): 1 × 10^7^ Huh7-vector or Huh7-sg SMPDL3A HCC cells were injected in the right axillary fossa of each mouse, respectively. After 16 days following subcutaneous inoculation, double-blinded evaluation was performed when the tumor size (V=π/6×L×W×W, L was the long diameter and W was the short diameter) was measured with a digital vernier caliper every 3 days. According to animal welfare ethical guidelines, tumor growth was not allowed to exceed 20 mm in diameter. All nude mice were subjected to *in vivo* imaging before euthanasia using isoflurane gas 4 weeks after the beginning of the measurement. Experimental mice were euthanized with an overdose of 2% pentobarbital sodium, and cervical spine dislocation was performed to confirm death. We only took one shot before the animals were sacrificed; the nude mice were placed in the same way when taking pictures. After saving the relevant data, the tumors were evaluated macroscopically and microscopically and were subjected to immunohistochemistry of Ki67 and PNCA.

All drug solutions were freshly prepared on the day of use. Experiments were performed under a project license (No:2018-L006) granted by the Institute Ethics Committee at the Affiliated Hospital of Nantong University, in compliance with the guidance of the care and use of laboratory animals issued by the Ministry of Science and Technology of the China.

### Co-Immunoprecipitation (Co-IP)

When the Huh7 cells overexpressing SMPDL3A had reached > 80% confluence, they were lysed with an appropriate amount of pre-chilled lysis buffer (NP-40, P0013F, purchased from Beyotime Biotechnology, China) and disrupted using ultrasound (SONICS VCX130PB). After centrifugation, the supernatant was collected for total protein extraction. Next, 400 ml Flag beads were added to the protein lysis solution, followed by incubation overnight at 4°C with rotation. After washing four times in Tris-buffered saline (TBS), an appropriate amount of 3 × Flag Peptide was added to the beads. Once the liquid in the tube was concentrated to a volume of < 50 µl, the concentrated samples were aspirated and added to equal volume of loading buffer for Western blotting after denaturation.

### Shotgun Mass Spectrometry

The protein solution or protein gel strip was digested with protease into a peptide mixture, and then separated by high performance liquid chromatography before importing into a high-resolution mass spectrometer for analysis in series. The peptide carried a charge after it had been ionized in the mass spectrometer. The mass-to-charge ratio (m/z) of each peptide was obtained through analysis by the detector. The mass spectrometer further bombarded the peptide ions to obtain the secondary mass spectrum signal. The relevant software (Proteome Discoverer 2.1, Thermo Fisher Scientific) package was used for searching, and the corresponding proteomic database (Uniprot_HomoSapiens_20386_20180905) was used to analyze the mass spectrometry data to restore the proteomic information in the samples. The mass spectrometry proteomics data have been deposited to the ProteomeXchange Consortium *via* the PRIDE partner repository with the dataset identifier PXD032824.

### Statistical Analysis

In this study, the SPSS 19.0 software package (IBM, Armonk, NY) was used for the analysis of experimental data. GraphPad Prism 7.0 software (GraphPad, San Diego, CA) was used to generate the graphs. All variables in the experimental data are presented as the mean ± standard deviation, and an independent sample t-test was used to compare the differences between groups. The correlation analysis between the expression level of SMPDL3A in HCC tissues and the clinical pathological data of patients with HCC was performed using chi-square test and t-test. Kaplan–Meier survival analysis was used to evaluate the effect of SMPDL3A expression on the 5-year tumor-free survival rate and overall survival of the patients. Univariate and multivariate analyses of the Cox proportional hazards regression model were used to analyze the risk factors related to the overall survival rate and tumor-free survival rate of patients with HCC after hepatectomy. P-values < 0.05 in all statistical data were considered to indicate a statistically significant difference.

## Results

### Expression Profiling Using Affymetrix GeneChip Microarrays to Identify DEGs After Knocking Down *VCAD1*

**Figure 1:**
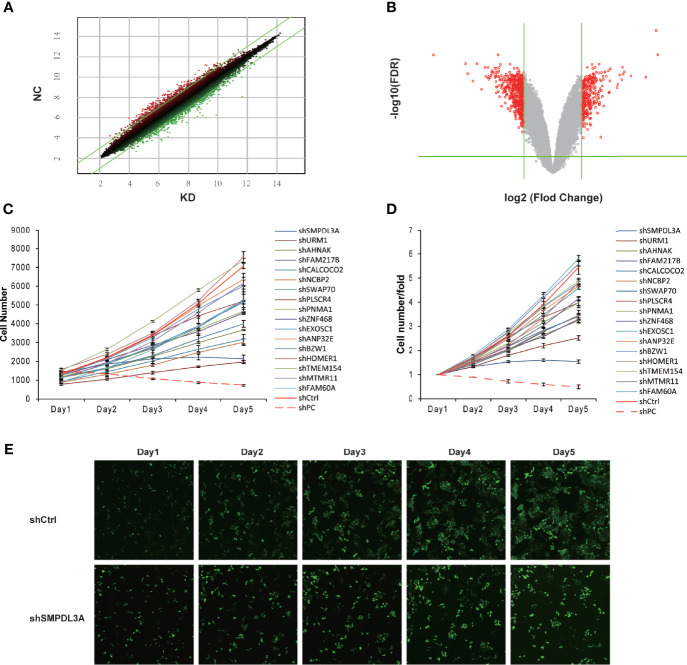
Differential gene expression screening from Affymetrix expression profile microarray after VDAC1 knockdown. **(A)** The scatter-plot of differential gene chip. **(B)** The Volcano Plot of differential expression profiles of gene. **(C, D)** Proliferation of corresponding differentially expressed genes was detected on the HCS platform. Knockdown of SMPDL3A significantly inhibits HCC cell proliferation. Positive control for PC, Negative control for Ctrl. **(E)** shSMPDL3A significantly suppressed proliferation.

To screen the downstream genes of *VDAC1* involved in the tumorigenesis and progression of HCC, we used Affymetrix GeneChip analysis for gene expression profiling to compare three *VDAC1-*knockout Huh7 cell lines (E5176-1, E5176-2, E5176-3) and three negative control Huh7 cell lines (E5175-1, E5175-2, E5175-3). **Figures 1A, B** show the scatter plot and volcano plot for genes with significantly different expression. Compared to the negative control cells, there were 205 upregulated mRNAs and 347 downregulated mRNAs in the shVDAC1 samples. Thirty-one representative DEGs were selected for further RT-qPCR verification (see **Table 2** for the primer information). Among them, 17 genes were consistent with the results of mRNA microarray (P < 0.05; **Table 3**). Using high-throughput screening, all 17 candidate genes were silenced in Huh7 cells, and the effect of gene knockdown on cell proliferation was compared. The cell proliferation of the shctrl group was normal and increased by 5.45-fold on the fifth day, compared to that on the first day. The cell proliferation of the shSMPDL3A cells was significantly reduced, and only increased by 1.54-fold on the fifth day compared to the first day. Knockdown of *SMPDL3A* and ubiquitin-related modifier 1 gene (*URM1*) suppressed the proliferation of HCC cell lines. Moreover, shSMPDL3A had the greatest inhibitory effect on cell proliferation (**Figures 1C–E**). Our research team has previously published a related study on *URM1* (6). In our follow-up research, we focus on the role of *SMPDL3A* in the tumorigenesis and progression of HCC.

**Table 2:** Primers designed to validate differentially expressed genes.

Target gene	Upstream Primer Sequence	Downstream Primer Sequence	Amplified Fragment (bp)
GAPDH	TGACTTCAACAGCGACACCCA	CACCCTGTTGCTGTAGCCAAA	121
TMEM154	GGAAGTGAAAACGTGAAAGTCCC	TCGGCATTTCTATTCATGCTGTT	110
ZNF532	GAACAGTGACCAGGGTATTGC	TGGAGACGACAGAAGGGATG	107
BZW1	AAGAGAGGTTTGACCCTACTCAG	CTGCATATCGACGGTAATCAAGT	132
PNMA1	GGTGTCCGATGTAGAAAAGA	CAGACGAATGACATAAGCAG	229
SMPDL3A	CTCACAGAGACAGCATTATGGTT	TTCACTGGTGTAACAGCAGGA	88
ANP32E	CCTTCTTGTTTCACTCCCTCC	ACCCCTGCTTTTCTTTCTTC	263
TBRG1	ATGGGGAAACTAATGCCTAACCT	AGGCTTCAAAGCTCATAGCTG	208
EDA2R	GGATTGCCAAGAAAATGAGTAC	CACGATTGATGACAGCACAGG	206
CTNNAL1	GTGTTTGAAGGAAGACGAGGAG	AGTCAGCGTCAGAGGTGAGC	169
CALCOCO2	TGAAGGAGGCGCAAGACAAAA	CATCTGCTGTTGCTCCAAGGT	154
URM1	AAGAAACATCGAGTCACTTTGC	GGTAGTCCAGCTCACCCAGTA	187
CCSER2	CCAGACCATGAAACATGATGCT	ATATCCTGTGGTGGCCCATTT	213
CNOT8	AGCCAGGTTATCTGTGAAG	CAAGTATTGATTCCAGAAGG	260
FAM60A	ATGGCTTCTGGTTCTAAC	AAGGCTTGAAGAGATGTG	139
PLSCR4	AGTTGGTGGTATCCATCCTGT	CCCTGGCATCCATGTTATTG	85
EXOSC1	GCGCCACCTGTGAGATACTG	CGGCAAGCGACGAAAAGATG	112
TP53RK	GGACTATGCTTCCAACTGCTT	GGTCTACTCCCTTATCCTCTGG	287
AHNAK	TGCCACCATCTACTTTGAC	GTTCTGGTCTTTGCATTCC	214
ZNF468	AGTCCCTCTCATCTCGCTTA	GAGAATTCTATGGCCACGTC	295
SWAP70	GCAGAAGAGGAAAAGAAACGC	GCTCCCGTACTCGCTGTAAAT	157
MTMR11	GAACACGATGGCTGGACTATG	GACACAATCAAGGAAGAGGAGAA	296
SPTY2D1	CACCTCGCTTTGTCTTCATC	CCTGGACTCTTAGCCTTATTGT	281
NCBP2	GAATCATTCGCACAGACTGG	CTGTGCCAGTTTTCCATAGC	137
SEL1L3	TGCGTCATACAATCTTGGAGTC	CATGTTTTGCCCATACAACAGC	219
NECTIN4	CCGTTCCTTCAAGCACTCCC	AGCCGTGTCCAGTTGTATGAG	276
MKNK2	CAGAAGAAACCAGCCGAACT	GTCTTCAAACCTGCCCGAGA	237
SRSF8	GGTCTCACTCGAAGTCTGGG	GGGAGGACTCCTGGTCATAG	143
MRPL19	GAGAAACGGCTGGATGATAGC	AGGCTCTTGTACTACTGGCTTC	96
SREK1IP1	ACCCTGGTCACCTGACTTTTG	TCAGTGGAACTGGATGAGTAAGA	244
HOMER1	CCGGAAAGTATCAACGGGACA	TCTGAGTTGGTTCAGCCCTTG	82
FAM217B	AAGCAAGACGCAAAAGGAAT	TGACCAGGGTGAAGATCAAAG	235

**Table 3:** qPCR expression of representative differentially expressed genes.

Gene	Average (2^-ΔΔCt^)		STDEV		p value (NC vs KD)
	NC	KD	NC	KD	
AHNAK*	1.000	0.352	0.032	0.027	0.0000
ANP32E*	1.000	0.494	0.036	0.039	0.0001
BZW1*	1.000	0.732	0.014	0.081	0.0049
CALCOCO2*	1.000	0.748	0.035	0.031	0.0007
EXOSC1*	1.001	0.713	0.040	0.047	0.0013
FAM60A*	1.002	0.479	0.072	0.020	0.0003
FAM217B*	1.003	0.569	0.092	0.010	0.0140
HOMER1*	1.001	0.879	0.048	0.015	0.0135
MTMR11*	1.002	0.599	0.076	0.062	0.0021
NCBP2*	1.001	0.872	0.039	0.059	0.0353
PLSCR4*	1.001	0.626	0.051	0.017	0.0003
PNMA1*	1.001	0.332	0.043	0.027	0.0000
SMPDL3A*	1.000	0.537	0.014	0.013	0.0000
SWAP70*	1.002	0.700	0.068	0.073	0.0063
TMEM154*	1.003	0.597	0.095	0.084	0.0053
URM1*	1.000	0.695	0.014	0.052	0.0006
ZNF468*	1.002	0.821	0.073	0.063	0.0321
CCSER2	1.003	1.694	0.089	0.069	0.0004
CNOT8	1.002	1.042	0.082	0.073	0.5667
CTNNAL1	1.001	1.435	0.058	0.077	0.0014
EDA2R	1.005	0.844	0.121	0.097	0.1471
MKNK2	1.001	1.243	0.047	0.056	0.0045
MRPL19	1.000	1.484	0.035	0.107	0.0017
NECTIN4	1.003	1.150	0.087	0.334	0.5001
SEL1L3	1.001	1.069	0.042	0.011	0.0510
SPTY2D1	1.001	1.383	0.043	0.057	0.0008
SREK1IP1	1.001	1.219	0.061	0.230	0.1876
SRSF8	1.001	1.986	0.042	0.028	0.0000
TBRG1	1.001	1.489	0.058	0.078	0.0010
TP53RK	1.000	1.637	0.039	0.069	0.0002
ZNF532	1.001	1.150	0.064	0.076	0.0585

*represents the gene whose qPCR result is consistent with the microarray result and p value is less than 0.05.

### Expression Profiling Using Affymetrix GeneChip Microarrays to Analyze the Genes With Significantly Different Expression After Knocking Down *SMPDL3A*

**Figure 2:**
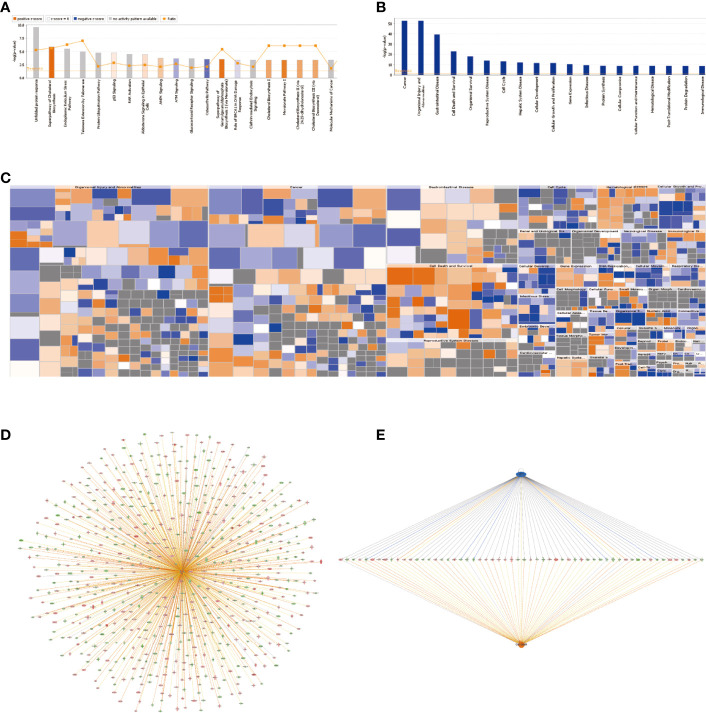
Expression profiling using Affymetrix GeneChip microarrays to analyze the genes with significantly different expression after knocking down SMPDL3A **(A)** Classic pathway analysis showed the significant enrichment of DEGs in the classic pathways. **(B)** The significant enrichment of DEGs in diseases and functions. The abscissa is the name of pathway, and the ordinate represents the degree of enrichment (logarithmic transformation at the base of 10) . **(C)** The “Diseases and functions” heatmap shows the activation/inhibition relationship of the changes in DEG expression on disease and function. Orange stands for activation (Z-score>0), blue for inhibition (Z-score<0), grey indicates not determined (Z-score unable to calculate). **(D)** Genetic disease and functional network diagram based on “Cell death”. Orange line indicates lead to activation, blue line indicates leads to inhibition, yellow lines indicate findings inconsistent with the state of molecules and gray lines indicate unknown. **(E)**The possible upstream regulatory networks and downstream functions of the DEGs. .

Affymetrix GeneChip analysis for gene expression profiling was used to study the knockdown effect of *SMPDL3A* in HCC cell lines, followed by bioinformatics analysis of the downstream genes with significant differential expression. Classic pathway analysis showed the significant enrichment of DEGs in the classic pathways (**Figure 2A**).

In this study, the “Superpathway of Cholesterol Biosynthesis” was significantly activated (Z-score = 2.887). **Figure 2B** shows the significant enrichment of DEGs in diseases and functions. Among them, the “Cancer” and “Organismal Injury and Abnormalities” pathways were significantly activated. The “Diseases and functions” heatmap shows the activation/inhibition relationship of the changes in DEG expression on disease and function (**Figure 2C**). The “Formation of vesicles” (Z-score = −2.529) and “Development of blastocyst” (Z-score = −2) were significantly inhibited, while the “Cell death” (Z-score = 2.074) and “Necrosis” (Z-score = 2.418) pathways were significantly activated. To study the activation and inhibition relationship between the genes and diseases or functions, we prepared a genetic disease or functional network diagram based on “Cell death” (**Figure 2D**). The network contains all DEGs related to “Cell death” and demonstrates the possible interaction and expression between them based on the Ingenuity database. **Figure 2E** shows the possible upstream regulatory networks and downstream functions. The consistency score is a measure of consistency and dense connections between the upstream regulatory factors, dataset, and “Diseases and functions” in the network, the higher the consistency score, the higher the accuracy of the results of regulatory effects.

### Relationship Between SMPDL3A Expression in HCC Tissues and the Prognosis of Patients With HCC

**Figure 3:**
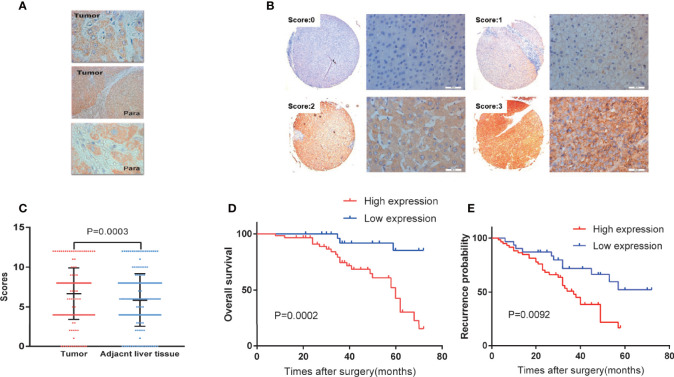
Analysis of SMPDL3A expression in human HCC tissues and survivals of HCC patients. **(A)** Expression of SMPDL3A in human HCC tissues and paracancerous tissues. SMPDL3A was mainly expressed in the nucleus or cytoplasm(upper tumor ×400, middle tumor and para ×40,down para ×400). **(B)** Staining scores of SMPDL3A protein expression in HCC tissue samples and surrounding noncancerous tissues. The staining score ranged from 0 to 3, with 0 for no staining, 1 for weak staining, 2 for moderate staining, and 3 for strong staining(×40, ×400). **(C)** The immunostaining score of SMDPL3A in 180 pairs of human HCC tissues and paracancerous tissues. Among the 180 pairs of tissue sections in this study, the difference in SMPDL3A expression in HCC tissues and the tumor-adjacent liver tissues were statistically significant (6.672±3.232 vs. 5.850±3.333, P = 0.0003). **(D)** Kaplan-Meier analysis of overall survival outcomes. The overall survival rates of patients in the high-SMPDL3A expression group at postoperative 1, 3, and 5 years(s) (96.61%,74.47% and 42.70%) were significantly lower than those in the low-SMPDL3A expression group (100%,91.83% and 85.27%, P=0.0002). **(E)** Kaplan-Meier analysis of disease-free survival outcomes. The 1-, 3-, and 5-year tumor-free survival rates (86.44%,50.94% and 16.56%) of the patients in the high-SMPDL3A expression group were significantly lower than those in the high-SMPDL3A expression group (90.32%,71.85% and 52.23%, P = 0.0092) .

Immunohistochemistry was used to detect the expression of SMPDL3A on tissue chips containing 180 pairs of HCC tissues and the tumor-adjacent liver tissues. SMPDL3A was mainly expressed in the nucleus and cytoplasm, showing as brown granular staining. Expression of SMPDL3A was different in the HCC tissue and the tumor-adjacent liver tissue (**Figure 3A**). The immunostaining score of SMDPL3A ranges from 0 to 12 points, and the product of the percentage of positively stained tumor cells and the staining intensity is used as the criterion. The expression level of SMDPL3A is defined as follows: staining intensity (no staining, 0 points; light staining, 1 point; moderate staining, 2 points; heavy staining, 3 points) (**Figure 3B**); the percentage of stained cells in the cell count (<5%, 0 points; 5 to 25%, 1 point; 26% to 50%, 2 points; 51% to 75%, 3 points; >75%, 4 points). The two points are multiplied to get the positive level. A total score greater than 6 is defined as a tissue with high expression of SMDPL3A, and a score less than or equal to 6 is defined as a tissue with low expression of SMDPL3A. Among the 180 pairs of tissue sections in this study, the difference in SMPDL3A expression in HCC tissues and the tumor-adjacent liver tissues were statistically significant (6.672 ± 3.232 vs. 5.850 ± 3.333, P = 0.0003, **Figure 3C**). According to the SMPDL3A expression in HCC tissues relative to the tumor-adjacent liver tissues of patients with HCC, we divided the patients into the high-SMPDL3A expression and the low-SMPDL3A expression groups. Combined to the clinicopathological data of the patients, the results of t-test and chi-square test showed that SMPDL3A expression was closely related to the vitamin K absence-II (PIVKA-II) level, liver cirrhosis, tumor diameter, microvascular invasion, and BCLC staging of the patients (P < 0.05, **Table 1**). Kaplan–Meier survival curves were additionally used to further analyze the relationship between SMPDL3A expression and survival of the patients. The overall survival rates of patients in the high-SMPDL3A expression group at postoperative 1, 3, and 5 years(s) were significantly lower than those in the low-SMPDL3A expression group (P = 0.0002; **Figure 3D**). Moreover, the 1-, 3-, and 5-year tumor-free survival rates of the patients in the high-SMPDL3A expression group were significantly lower than those in the high-SMPDL3A expression group (P = 0.0092; **Figure 3E**). Univariate and multivariate analyses of the Cox proportional hazard regression model were used to analyze the risk factors related to the overall survival rate and tumor-free survival rate of patients with HCC after hepatectomy. The results suggest that preoperative alpha-fetoprotein level, liver cirrhosis, Child–Pugh score, portal venin tumor thrombus, microvascular invasion, and molecular level of SMPDL3A were independent risk factors affecting the overall survival of patients with HCC (**Table 4**). The SMPDL3A level was an independent risk factor affecting postoperative tumor-free survival of patients with HCC (**Table 5**).

**Table 4:** Univariate and multivariate analyses of independent risk factors for overall survival of patients with hepatocellular carcinoma after hepatectomy.

Clinical Valuable	Univariate analysis		Multivariate analysis	
	HR(95% CI)	p-value	HR(95% CI)	p-value
Gender	1.372 (0.620, 3.307)	0.435		
AFP	2.565 (1.279, 5.145)	0.008	2.498 (1.260, 4.952)	0.009
Tumor number	1.145 (0.381, 3.440)	0.810		
Cirrhosis	0.527 (0.258, 1.074)	0.078	0.526 (0.261, 1.058)	0.072
Child-pugh	0.449 (0.200, 1.004)	0.051	0.439 (0.197, 0.979)	0.044
PVTT	0.256 (0.079, 0.826)	0.023	0.244(0.077, 0.772)	0.016
MVI	2.813 (1.003, 7.884)	0.049	2.898 (1.034, 8.117)	0.043
SMPDL3A	0.537 (0.283, 1.019)	0.057	0.524 (0.277, 0.990)	0.047

MVI, microvascular invasion; PVTT, portal vein tumor thrombus; AFP, α-fetoprotein; HR, Hazard Ratio.

**Table 5:** Univariate and multivariate analyses of independent risk factors for tumor-free survival of patients with hepatocellular carcinoma after hepatectomy.

Clinical Valuable	Univariate analysis		Multivariate analysis	
	HR(95% CI)	p-value	HR(95% CI)	p-value
Gender	0.564 (0.173, 1.842)	0.343		
AFP	2.374 (0.900, 6.260)	0.080	1.717 (0.797, 3.698)	0.167
Tumor number	1.772 (0.373, 8.408)	0.472		
Cirrhosis	0.858 (0.325, 2.270)	0.758		
Child-pugh	2.442 (0.730, 8.169)	0.147		
PVTT	0.400 (0.094, 1.707)	0.216		
MVI	0.885 (0.286, 2.739)	0.049	0.638 (0.224, 1.812)	0.398
SMPDL3A	0.146 (0.043, 0.501)	0.002	0.156 (0.046, 0.529)	0.003

MVI, microvascular invasion; PVTT, portal vein tumor thrombus; AFP, α-fetoprotein; HR, Hazard Ratio.

### Establishment of a Crispr-Cas9 Lentiviral Vector to Knockout *SMPDL3A*

**Figure 4:**
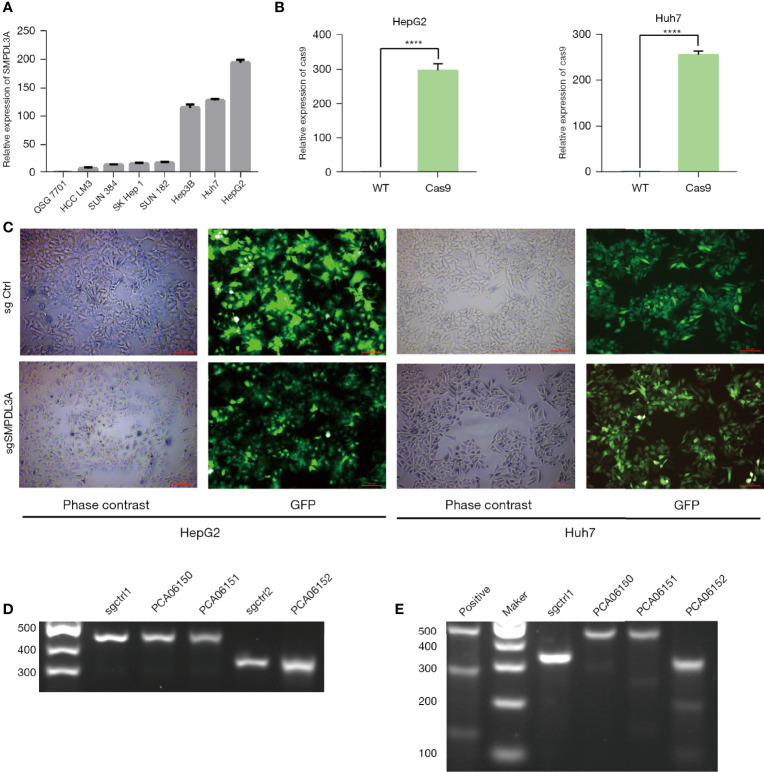
The CRISPR/Cas9 dual-vector lentivirus knocked out the target sequence of theSMPLD3A gene. **(A)** SMPDL3A expression levels in HCC cell lines and normal liver cell line. **(B)** CAS9 overexpression was confirmed by QPCR assays in HepG2 and Huh7 cell lines (****P<0.0001). **(C)**Fluorescence microscopy images taken under white light exposure and excitation of green light. Infection efficiency was almost 100%. **(D)** The agarose gel electrophoresis figure of DNA hybridization products. sgctrl1 and sgctrl2 were used as negative control. **(E)** The agarose gel electrophoresis figure of after T7E1 digested . The expected positions of DNA bands cleaved by T7E1in the samples of . PCA06151and PCA06152. sgctrl1 was used as negative control, positive was used as positive control.

To observe the effect of SMPDL3A on the progression of HCC, we used RT-qPCR to detect the background expression of SMPLD3A in eight common HCC cell lines (**Figure 4A**). Among them, HepG2 and Huh7 had the highest SMPLD3A expression. For this reason, we selected these two cell lines for subsequent experiments. The Crispr-Cas9 lentiviral vector, through two lentiviruses, was used to separately introduce the Cas9 protein- and sgSMPDL3A-sequence expression cassettes into the cells to achieve target gene knockout. The Lenti-Cas9 lentiviral vector was first transfected with the HepG2 and Huh7 cell lines. Our results showed that Cas9 was overexpressed 300-fold in the HepG2 cells and 260-fold in the Huh7 cells, suggesting that Cas9 was successfully introduced into HCC cell lines, and subsequent experiments could be proceeded (**Figure 4B**). Three sgSMPDL3A lentiviruses [PCA06150 (sgRNA sequence: 5′-ACAGTGTCTGTTGAGAGTTC-3′), PCA06151 (sgRNA sequence: 5′-TGGAAAGAGACTCTGGATGG-3′), and PCA06152 (sgRNA sequence: 5′-AGACTcGATGATCCTAAGGTT-3′)] were used to independently transfect the HepG2 and Huh7 cell lines carrying Cas9. The successfully transfected cells were puromycin resistant, while the untransfected cells died; therefore, the cells with stable knockout of *SMPDL3A* were selected (**Figure 4C**). The Surveyor assay was used to confirm the cutting effect. After harvesting the transfected cells for genomic DNA extraction, the designed primers around the sgSMPDL3A binding site were used for PCR amplification and annealing to obtain hybrid DNA products, which were then separated by agarose gel electrophoresis (**Figure 4D**). The DNA products were subjected to T7 endonuclease I(T7E1)digestion, followed by separating the fragments by agarose gel electrophoresis (**Figure 4E**). Compared to the control and PCA06150 groups, the PCA06151 and PCA06152 groups had DNA fragments at the expected position in the agarose gel, indicating that they had sgSMPLD3A activity. Among these two groups, the DNA fragment in the agarose gel showed higher intensity under UV light detection. Thus, PCA06152 was selected for subsequent experiments.

### SMPDL3A Promoted the Proliferation and Migration and Inhibited Apoptosis of HCC Cells *In Vitro*

**Figure 5:**
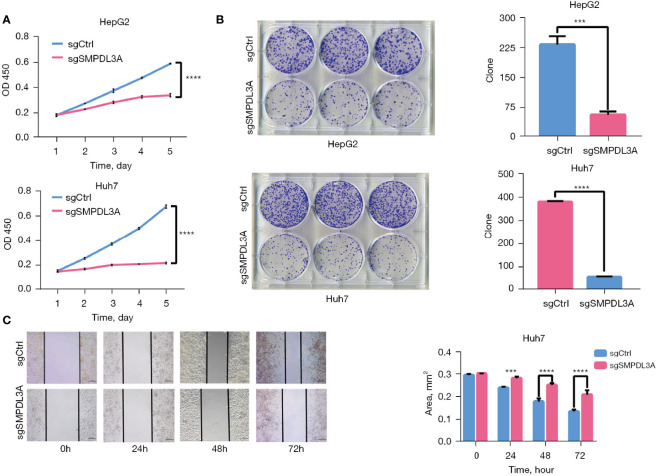
SMPDL3A promotes HCC cell proliferation and migration *in vitro*. **(A)** The proliferation of HepG2 and Huh7 cells in the SMPDL3A knockout and control groups. SMPDL3A knockout HCC cell line proliferation was significantly lower than that in the control group (****P<0.0001). **(B)** The number of clones of HepG2 and Huh7 cells sgSMPDL3A was significantly lower than that in the control group (***P=0.0002, ****P<0.0001). **(C)** Huh7 cell wound healing (scratches) in the sgSMPDL3A and control groups at 0, 24, 48, and 72h (***P=0.0005, ****P<0.0001).

**Figure 6:**
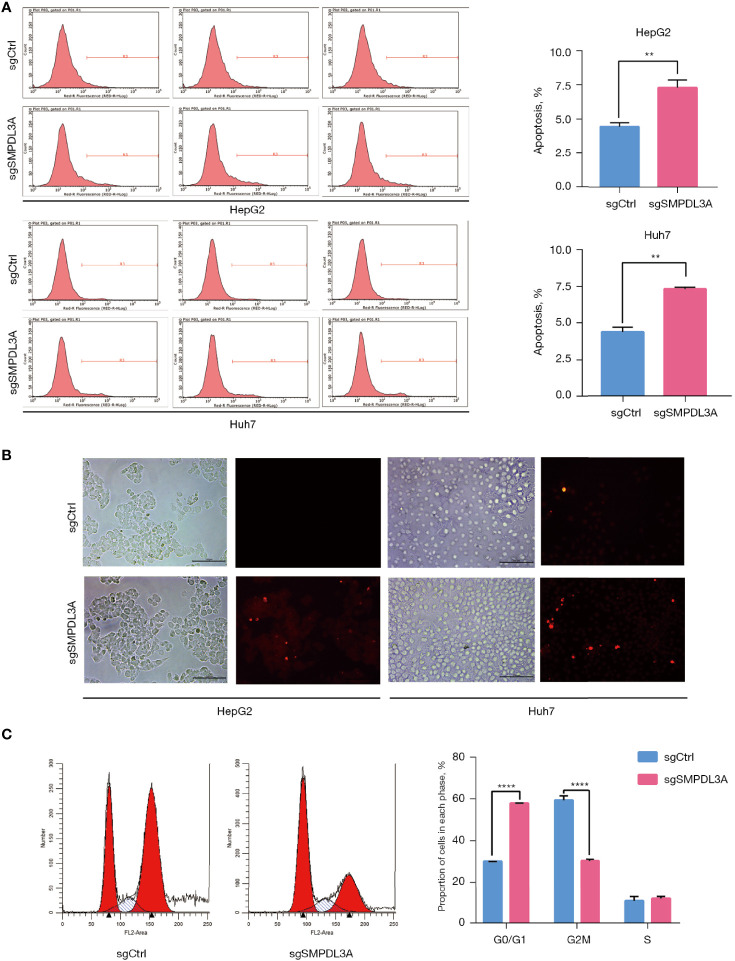
The effect of SMPDL3A on the cycle and apoptosis. **(A)** Apoptotic cells were detected by flow cytometry (**P=0.01). **(B)** Fluorescence field of HepG2 and Huh7 cells in the SMPDL3A knockout and control groups; apoptotic cells are marked by red fluorescence. The proportion of apoptotic cells in the sgSMPDL3A group was significantly higher than that in the control group. **(C)** Cell cycle distribution was analyzed by flow cytometry the cells in the early DNA synthesis phage (G0 /G1) of the sgSMPDL3A group were significantly increased, while the cells in the late DNA synthesis phase (G2 phase) were significantly reduced (****P<0.0001).

Through CCK-8 cell growth curve analysis (**Figure 5A**) and clonogenic assay (**Figure 5B**), sgSMPDL3A was confirmed to inhibit the proliferation and tumorigenesis of HepG2 and Huh7 HCC cell lines. In addition, the role of SMPDL3A in cell migration was evaluated using an *in vitro* scratch assay. The results showed that sgSMPDL3A inhibited the migration ability of HCC cells (**Figure 5C**). Through flow cytometry and TUNEL staining, sgSMPDL3A was found to promote the apoptosis of HCC cells (**Figures 6A, B**). By studying the cell cycles of the two groups of cells, the number of cells in the sgSMPDL3A group in the early stage of DNA synthesis (G0/G1 phase) was significantly higher than that in the control group, while the number of cells in the late stage of DNA synthesis (G2 phase) was significantly reduced (****P<0.0001). The proliferation index (PI = the sum of the proportion of cells in S phase and G2 phase/the sum of the proportion of cells in G1 phase, S phase and G2 phase) of sgSMPDL3A was 0.393, which was significantly lower than 0.700 in the control group (**Figure 6C**). The proliferation index of sgSMPDL3A was 0.393, which was significantly lower than the index of 0.700 in the control group.

### SMPDL3A Promoted Tumor Growth *In Vivo*

**Figure 7:**
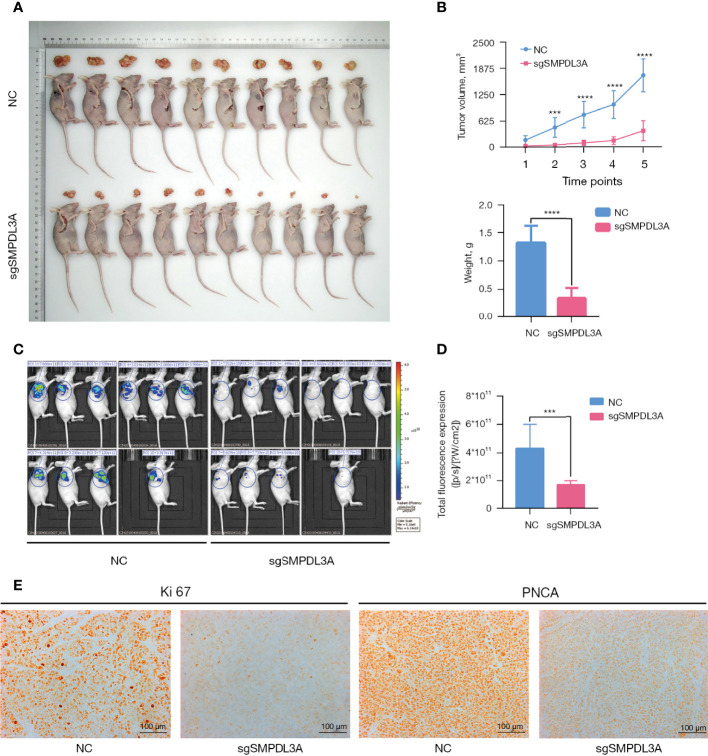
The effect of SMPLD3A on subcutaneous tumor formation in a nude mouse xenograft model of liver cancer. **(A)** Representative images of tumors in the SMPDL3A knockout and the control groups. **(B)** sgSMPDL3A led to a significant decrease in tumor volume and weight, compared with control group (****P=0.0001). **(C)** Fluorescence images of each group in nude mice subcutaneous tumor. **(D)** Fluorescence intensity of two groups of nude mice subcutaneously implanted tumors (***P=0.001). **(E)** Representative images of Ki67 and PNCA staining of subcutaneous tumors in each group.

To further explore whether SMPDL3A promoted the proliferation of HCC cell lines *in vivo*, a tumor-bearing mouse model generated through subcutaneous inoculation of HCC cells was established in BALB/c nude mice. Compared to the control group, the tumor growth rate of the sgSMPDL3A group was significantly lower (**Figure 7A**), and the weight of subcutaneous xenograft tumor in the sgSMPDL3A nude mice was significantly less than that of the control group (0.341 ± 0.184g *vs*. 1.314 ± 0.306 g, P < 0.001) (**Figure 7B**). The tumor fluorescence signal in the nude mice was observed using a small-animal live imaging system, and the results showed that the fluorescence expression of the sgSMPLD3A group was significantly lower than that of the control group (P = 0.0001) (**Figures 7C, D**). Immunohistochemistry of Ki67 and PNCA was performed to compare the protein expression in the two subcutaneous xenograft tumor-derived groups of HCC cells. The results showed that the expression of Ki67 and PCNA in the subcutaneous xenograft tumors of the control group was higher than that of the sgSMPDL3A group (**Figure 7E**).

### Enhancer of Rudimentary Homolog (ERH) Interacted With SMPLD3A

**Figure 8:**
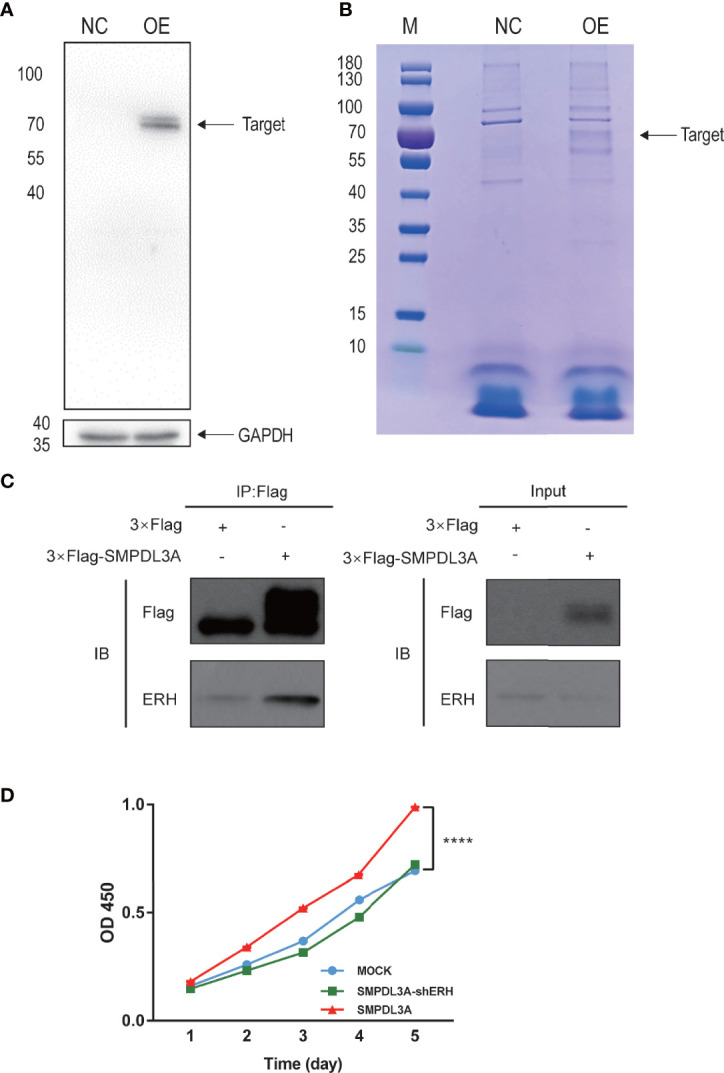
Interactions between SMPDL3A and ERH. **(A)** The liver cancer cells overexpressing SMPDL3A were successfully detected by western blotting. **(B)** Coomassie brilliant blue staining, indicating the presence of a 72-kDa protein band in the protein gel. **(C)** Verification of the interaction between SMPDL3A and ERH by Co-Immunoprecipitation (Co-IP) assay. **(D)** The function of SMPDL3A affecting the proliferation of Huh7 is regulated by ERH and verified by CCK8 (****P<0.0001).

To identify SMPDL3A-related proteins in HCC, the Huh7 cell line with a Flag tag and overexpressing SMPDL3A was constructed. The cells overexpressing SMPDL3A were successfully detected by western blotting (**Figure 8A**). After extracting the total protein of the experimental and control groups, the protein interacting with SMPDL3A was affinity purified by co-IP. The purified protein complex was subjected to SDS-PAGE and Coomassie brilliant blue staining, indicating the presence of a 72-kDa protein band in the protein gel (**Figure 8B**). The extracted protein band analyzed by Shotgun mass spectrometry for the identification of a large number of proteins (**Table 6**) showed that the coverage of ERH was higher. A literature review of related studies indicated that ERH played an important role in the tumorigenesis and progression of HCC (7). Hence, ERH was selected as the target molecule for the subsequent experiments. Co-IP was used to further verify the effect between ERH and SMPDL3A. The Flag tag was successfully detected in the over expressed (OE) group in Input, indicating that 3 × Flag-SMPDL3A was successfully overexpressed in the target cells. In IP, both NC and OE groups were detected with the target band. The OE group showed a significant increase in the expression of the interacting protein compared to the NC group, i.e., increased expression = interacting protein(s), indicating that the 3 × Flag-target gene protein pulled down the interacting protein and that there was an interaction between the proteins (**Figure 8C**). To verify whether SMPDL3A affected the proliferation of HCC cells by regulating ERH, a Huh7 cell line overexpressing SMPDL3A (SMPDL3A cells) and another Huh7 cell line overexpressing SMPDL3A with knockdown of ERH (SMPDL3A-shERH cells) were established. The cell viability assay using CCK-8 kit was performed on days 1, 2, 3, 4, and 5 (**Figure 8D**). The results showed that the proliferation ability of the SMPDL3A cells was significantly higher than that of the cells in the control group (MOCK). The proliferation ability of the SMPDL3A-shERH cells was similar to that of the control cells. These results indicated that the knockdown of ERH significantly inhibited the proliferation of HCC cells, confirming that the function of SMPDL3A was regulated by ERH.

**Table 6:** MS analysis of SMPDL3A-associated proteins.

Gene Name	Coverage [%]	Peptides	PSMs
SPTBN1	4	9	10
PRMT5	3	2	6
P4HB	2	1	1
MYH9	0	1	1
HSPA5	5	2	2
HSP90AB1	1	1	1
HNRNPA1	2	1	1
FLNA	1	2	2
FGG	2	1	1
ERH	11	1	2
EIF4B	12	5	5
CASP14	5	1	1
BCLAF1	2	2	2

## Discussion

HCC is a common malignant tumor with high mortality worldwide. The underlying mechanism of the tumorigenesis and tumor progression of HCC is complicated, and the specific molecular mechanisms are still not fully understood. Thus, further exploration of new functional genes in HCC and their molecular mechanisms affecting tumorigenesis and tumor progression may provide important guidance for revealing the pathogenesis of HCC to develop new therapeutic drugs to improve patient prognosis. Our previous study showed that the expression of VDAC1 is closely related to the progression of HCC (4). VDAC1 is a 31-kDa pore-forming protein found on the outer mitochondrial membrane of all eukaryotic cells (8). The mitochondrial outer membrane channel composed of VDAC1 regulates the release of many apoptotic genes and molecules, such as cytochrome C, apoptosis inducing factor (AIF), serine peptidase 2 (HTRA1), and endonuclease G (Endo G). Overexpression of VDAC1 in many types of tumors may be involved in some activities of tumor cells with active metabolism and high energy consumption (9). VDAC1 is a potential target for regulating apoptosis, both inducing apoptosis in malignant tumor cells and inhibiting apoptosis in neurogenerative diseases (10). The tumorigenesis and tumor progression of HCC are complex processes, and the specific mechanism by which VDAC1 alters HCC metabolism has not yet been elucidated.

To further study the downstream related molecules of VDAC1, expression profiling using Affymetrix GeneChip microarrays and high-throughput functional screening based on cell proliferation was used to confirm that SMPDL3A promoted the proliferation of HCC cells. However, the mechanisms and related molecules regulating between VDAC1 and SMPDL3A needs more study. SMPDL3A is a member of the acid sphingomyelinase (aSMase) family. SMPDL3A and aSMase share many key sequences and similar structural characteristics, including metal phosphodiesterase domains and catalytic residues, Zn^2+^ binding sites, distinctive C-terminal domains, and overall three-dimensional structure of calcineurin (11). aSMase is an important enzyme in sphingolipid metabolism that plays an essential role in apoptosis, immunity, development, and tumor growth (12–15). Studies have shown that SMPDL3A has the activity of nucleotide phosphodiesterase and hydrolyzes the phosphate from the end of nucleotide triphosphates, such as ATP and nucleotide derivatives, including cytidine diphosphate (CDP)-ethanolamine, CDP-choline, adenosine diphosphate-ribose, and nucleotide diphosphate (16). SMPDL3A also plays an important role in aspects including cell processing, transportation, and secretion. Liver X receptor (LXR) agonists and cholesterol load increase the expression of SMPDL3A, indicating that it plays an important role in oxysterols and lipid metabolism (17). SMPDL3A also has phosphoramidate activity, which may be related to the anabolism of phosphoramidate prodrugs in the liver (18). In addition, SMPDL3A may affect cell differentiation, and SMPDL3A expression inhibits the reprogramming of mouse embryonic fibroblasts into non-dividing pluripotent stem cells (19). In addition, SMPDL3A may be involved in immunomodulation, such as by affecting the secretion of interleukin (IL)−6 in mouse macrophages (20). Moreover, SMPDL3A is upregulated in white blood cells of children with sepsis (21), in megakaryocytes in a murine model of sepsis (22), and in canine pyrometra (23). However, few studies on SMPDL3A in tumors have been reported. A study has shown that SMPDL3A is highly expressed in bladder cancer and directly interacts with a tumor suppressor gene, i.e., deleted in bladder cancer chromosome region candidate 1 (*DBCCR1***)** (24) SMPDL3A is also used as an indicator to evaluate the prognosis of colon cancer and may become a target for the diagnosis and treatment of colon cancer in the future (25). Nevertheless, no previous report has examined the role of SMPDL3A in HCC. This study used a SMPDLA molecule as the research object to further analyze its expression in HCC, its relationship with clinicopathological indicators of HCC, and its relationship between long-term survival after hepatectomy for HCC. Subsequently, we tested the expression level of SMPDL3A using microarray to reveal the differential expression of SMPDL3A between 180 pairs of HCC tissues and the tumor-adjacent liver tissues. We also analyzed the relationship between SMPDL3A and clinical indicators and showed that SMPDL3A expression was closely related to PIVKA-II level, liver cirrhosis, tumor diameter, microvascular invasion, and BCLC staging. Patients with high SMPDL3A expression had significantly lower 1-, 3-, and 5-year overall survival and tumor-free survival rates than patients with low SMPDL3A expression. The molecular level of SMPDL3A were independent risk factors affecting the overall survival and tumor-free survival of patients with HCC. Thus, it is of great significance to perform an in-depth study on the role and mechanism of SMPDL3A in the tumorigenesis and tumor progression of HCC.

In the current study, we tested the relative expression of SMPDL3A in several common HCC cell lines and selected HepG2 and Huh7 cells, which had higher SMPDL3A expression compared to the other cell lines, for the follow-up research. Further application of CRISPR/Cas9 lentivirus vectors was used to knockout *SMPDL3A* in HepG2 and Huh7 cell lines to observe any changes in the biological function in HCC after knocking out *SMPDL3A*. Our results showed that the proliferation and tumorigenesis of HepG2 and Huh7 cells were significantly reduced after knocking out *SMPDL3A*. To further explore the reason for the decline in the cell proliferation through studying the cell cycle, we showed that knocking out *SMPDL3A* significantly prolonged early DNA synthesis and reduced late DNA synthesis in HCC cells. Moreover, the proliferation index of the sgSMDPL3A group (0.393) was significantly lower than that of the control group (0.700). This explains why the proliferation ability of hepatoma cells in the sgSMPDL3A group was significantly lower than that in the control group. In addition, through flow cytometry and TUNEL staining, we showed that the apoptosis of the HepG2 and Huh7 cell lines in the sgSMPDL3A group was significantly higher than that of the control group. In tumor-bearing nude mice, the tumor weight, fluorescence expression, and tumor diameter of the nude mice in the sgSMPDL3A group were lower than those in the control group. In immunohistochemistry, the expression of proliferation-related molecules Ki67 and PNCA in the sgSMPDL3A group was significantly lower than that in the control group. These results indicated that SMPDL3A promoted tumor proliferation in HCC, and that the inhibition of SMPDL3A expression suppressed tumor proliferation and promoted apoptosis. Thus, SMPDL3A may be a novel target for tumor-targeted therapy.

The specific molecular mechanism of SMPDL3A in promoting proliferation and inhibiting apoptosis of HCC remains unclear. In the current study, we used mass spectrometry to screen the proteins that may interact with SMPDL3A. In our IP study, the ERH of OE group was significantly higher than that of NC group. IP process was successful. In our future work, we will complete IP with anti-ERH antibody and then blotted with Flag. It was further verified the interactions between SMPDL3A and ERH. ERH is essential for the expression of multiple genes associated with the cell cycle and DNA damage response. ERH is highly expressed in breast cancer tissues (26), ovarian cancer tissues (27), and HCC tissues (7), and affects both migration and tumorigenicity. Knockdown of *ERH* inhibits the migration and proliferation of bladder cancer cells (28) and ovarian cancer cells (27). A previous study has also shown that ultraviolet light enhances the DNA damage of HepG2 cells after knockdown of *ERH*. ERH regulates the splicing of DNA damage response protein, i.e., ataxia telangiectasia and Rad3-related protein, in HCC cells and targets the DNA damage response by Chk1 inhibitors to enhance chemotherapy against HCC cells (7). Inhibition of ERH expression slows down proliferation, promotes apoptosis, and inhibits metastasis and invasion by regulating the epithelial-mesenchymal transition of SKOV3 cells. The cell viability assay verified that ERH regulated the functions of SMPDL3A in HCC cells. However, the mechanisms of these effects are not clear and merit further exploration. In our follow-up studies we will investigate the domain of the interaction between ERH and SMPDL3A.

To conclude, our findings showed that SMPDL3A, as an important proto-oncogene, regulated apoptosis of HCC cells by interacting with ERH and promoting the proliferation of HCC cells. More importantly, we discovered the biological role of *SMPDL3A* in HCC, and found that high SMPDL3A expression in HCC may lead to poor prognosis. Our results provide a broader direction for further research of *SMPDL3A* in cancer.

## Data Availability Statement

The datasets presented in this study can be found in online repositories. The names of the repository/repositories and accession number(s) can be found below: GEO, GSE198538, GSE198537 & ProteomeXchange *via* the PRIDE database with identifier PXD032824.

## Ethics Statement

The studies involving human participants were reviewed and approved by Institute Ethics Committee at the Affiliated Hospital of Nantong University. The patients/participants provided their written informed consent to participate in this study. The animal study was reviewed and approved by Institute Ethics Committee at the Affiliated Hospital of Nantong University.

## Author Contributions

Conception and design, all authors. Administrative support, ZC. Provision of study materials or patients, YZ, WC, XC, FW, CG, FS, FLS, XL, and WF. Collection and assembly of data, YZ and XC. Data analysis and interpretation, YZ and WC. Manuscript writing, all authors. Final approval of manuscript, all authors.

## Funding

This study was supported by grants from the National Natural Science Foundation of China (NO. 81871927), the Jiangsu Science and Technology Department Project (BL2014060) and the Nantong Hepatobiliary and Pancreatic Surgery Disease Research Center Construction Project (HS2015001).

## Conflict of Interest

The authors declare that the research was conducted in the absence of any commercial or financial relationships that could be construed as a potential conflict of interest.

## Publisher’s Note

All claims expressed in this article are solely those of the authors and do not necessarily represent those of their affiliated organizations, or those of the publisher, the editors and the reviewers. Any product that may be evaluated in this article, or claim that may be made by its manufacturer, is not guaranteed or endorsed by the publisher.
