# Supplementary figures and images for: Sphingomyelin Phodiesterase Acid-Like 3A Promotes Hepatocellular Carcinoma Growth Through the Enhancer of Rudimentary Homolog
Source: Front Oncol. 2022 May 24;12:852765. doi: 10.3389/fonc.2022.852765 (PMC9171240; doi:10.3389/fonc.2022.852765)

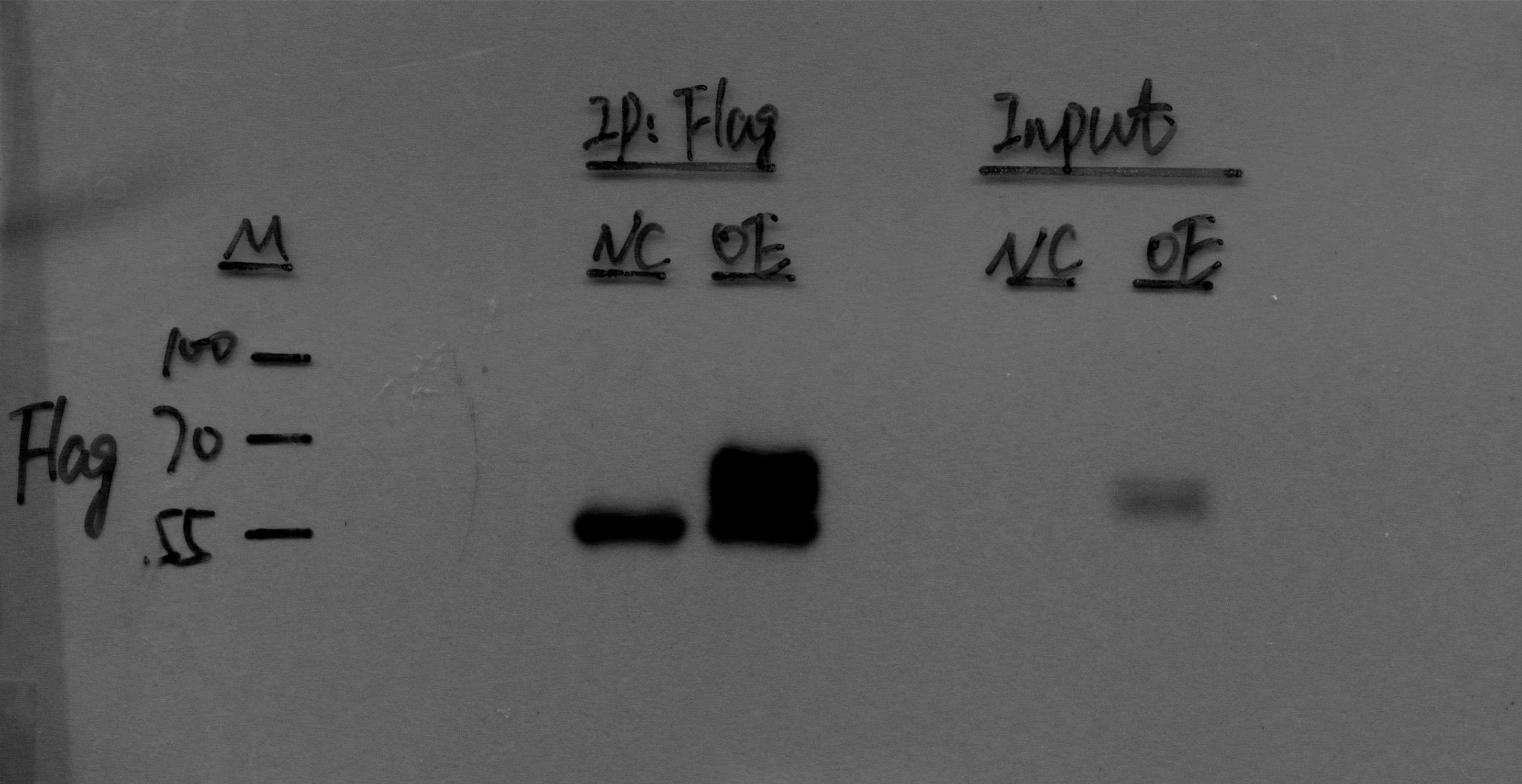

Supplement: Supplementary file 1 [file Presentation_1.zip › co-ip Input.JPEG]

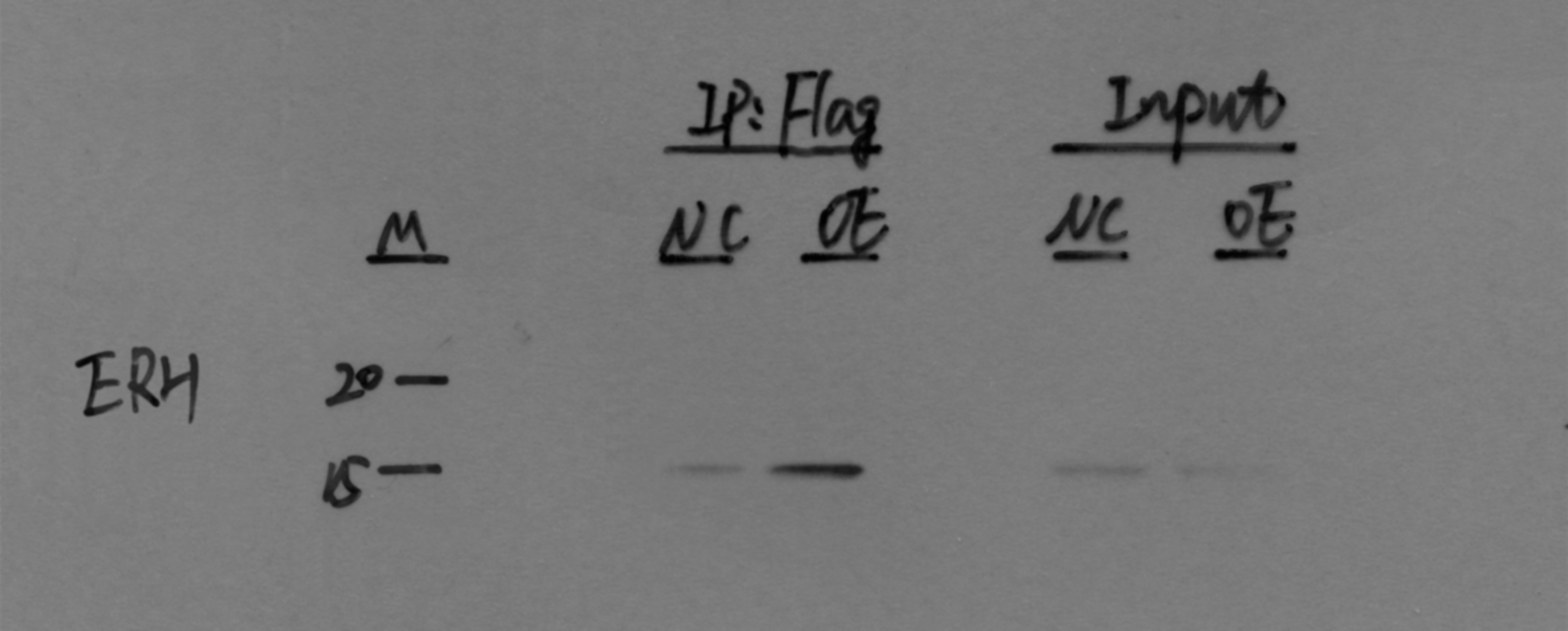

Supplement: Supplementary file 1 [file Presentation_1.zip › co-ip IP.JPEG]

## Slide 1
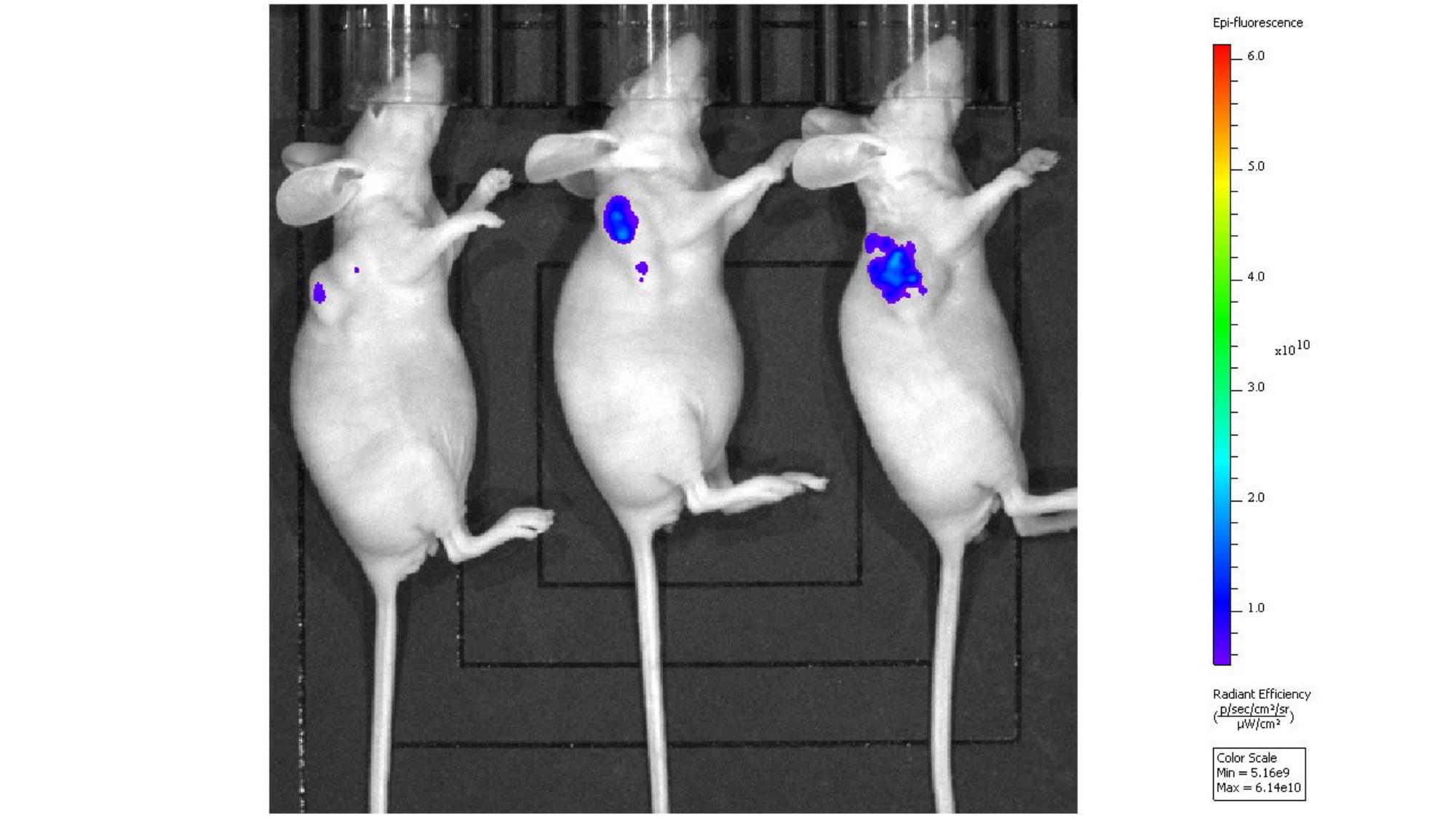

## Slide 2
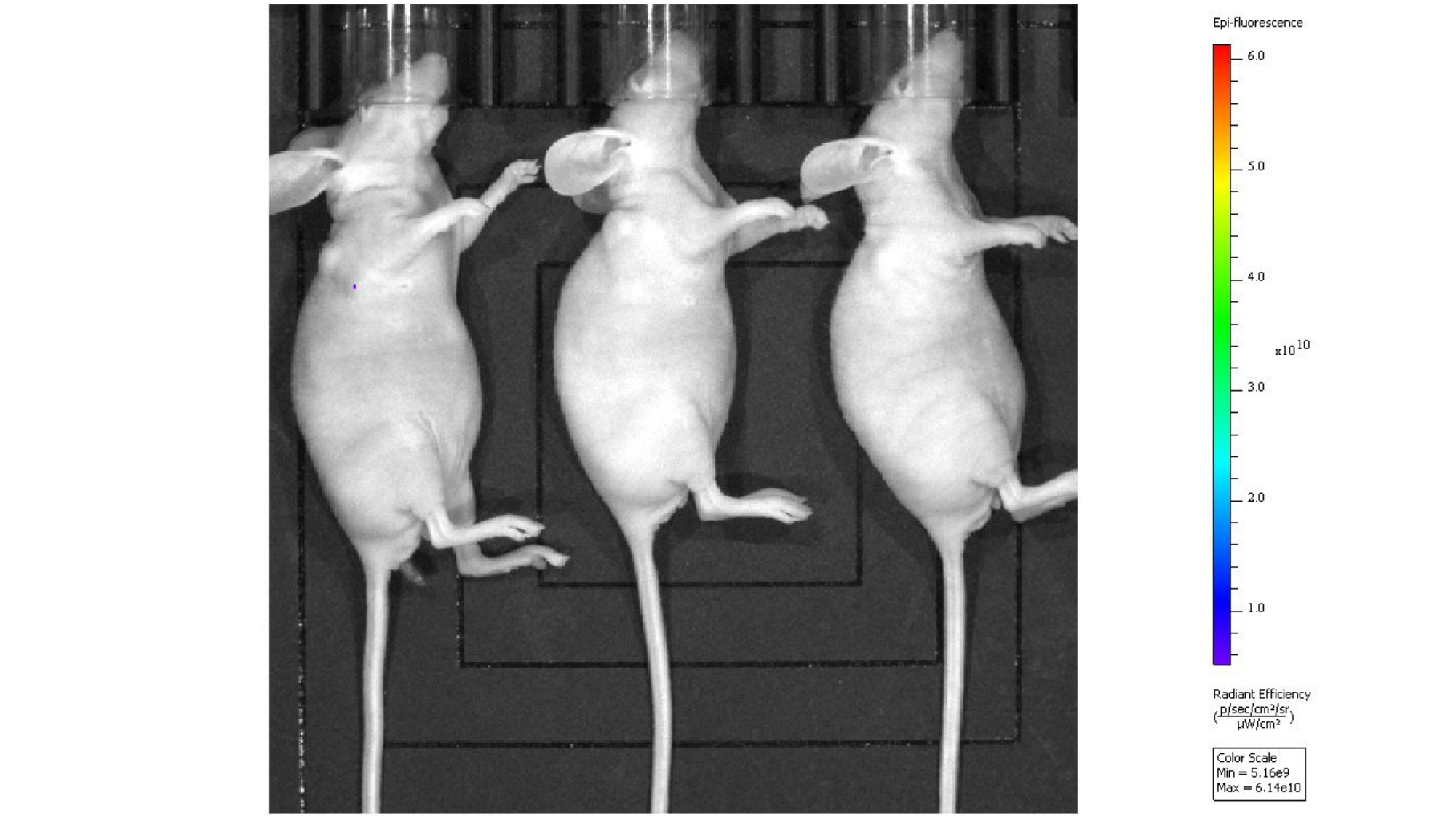

## Slide 3
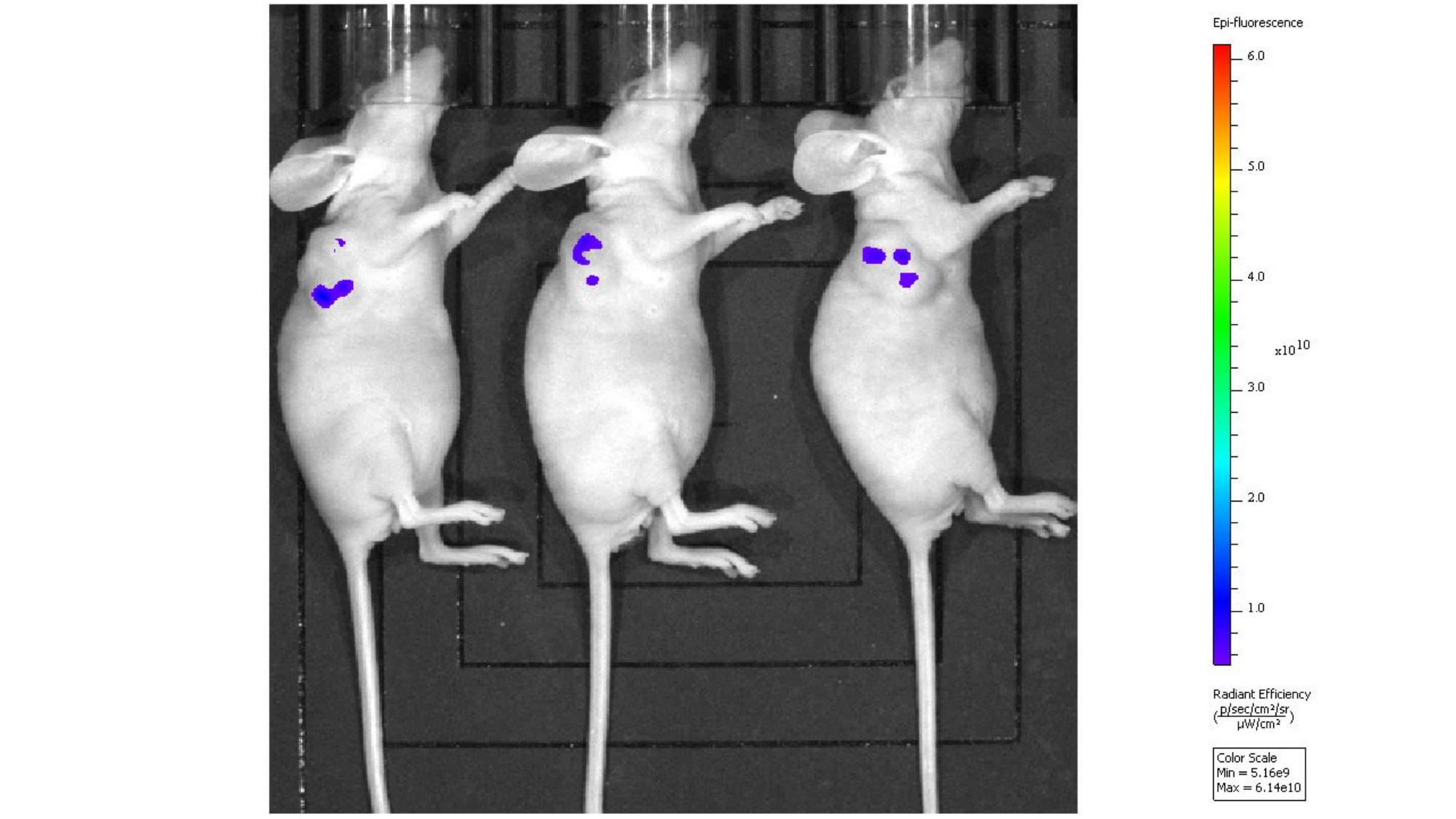

## Slide 4
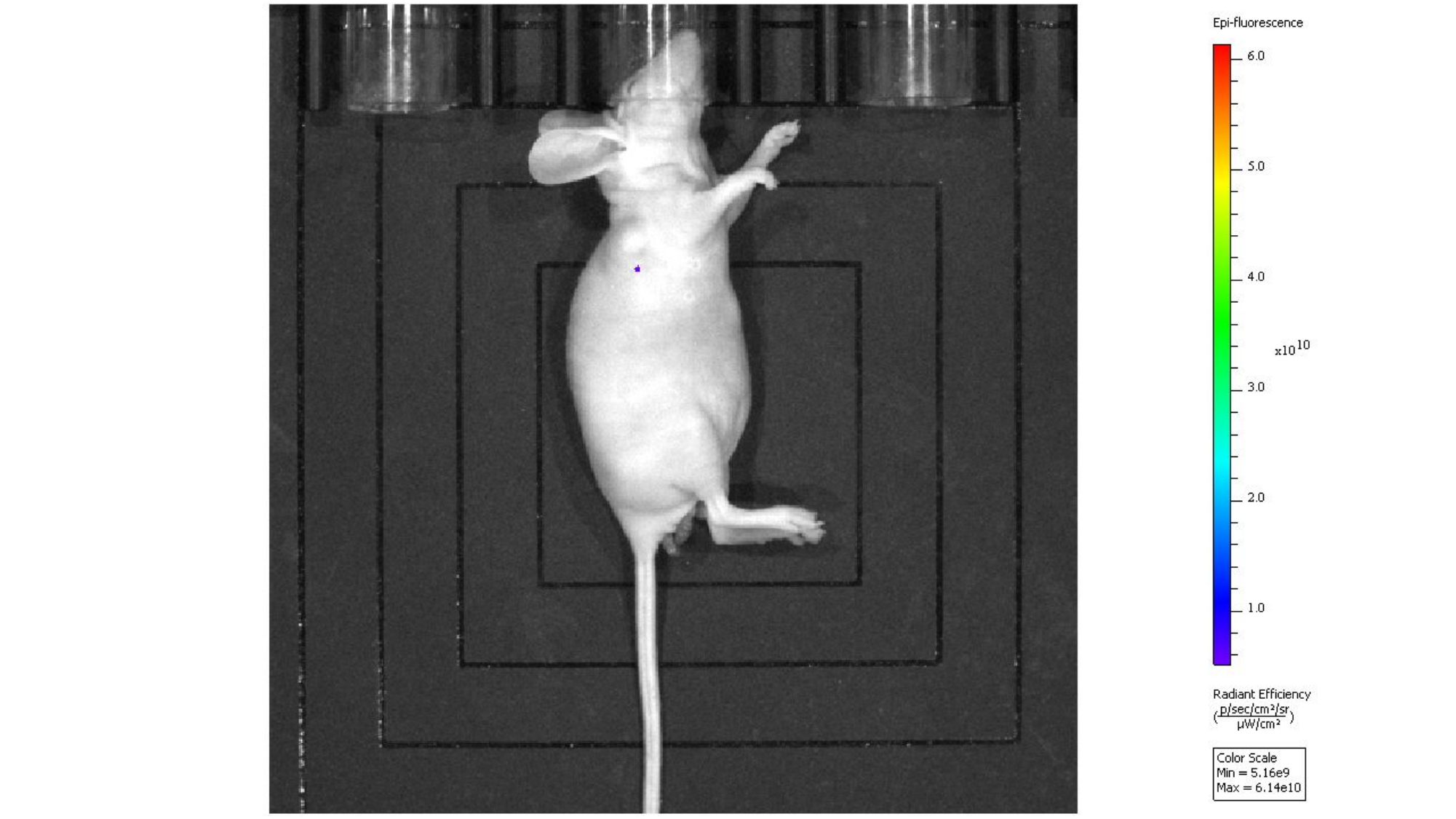

## Slide 5
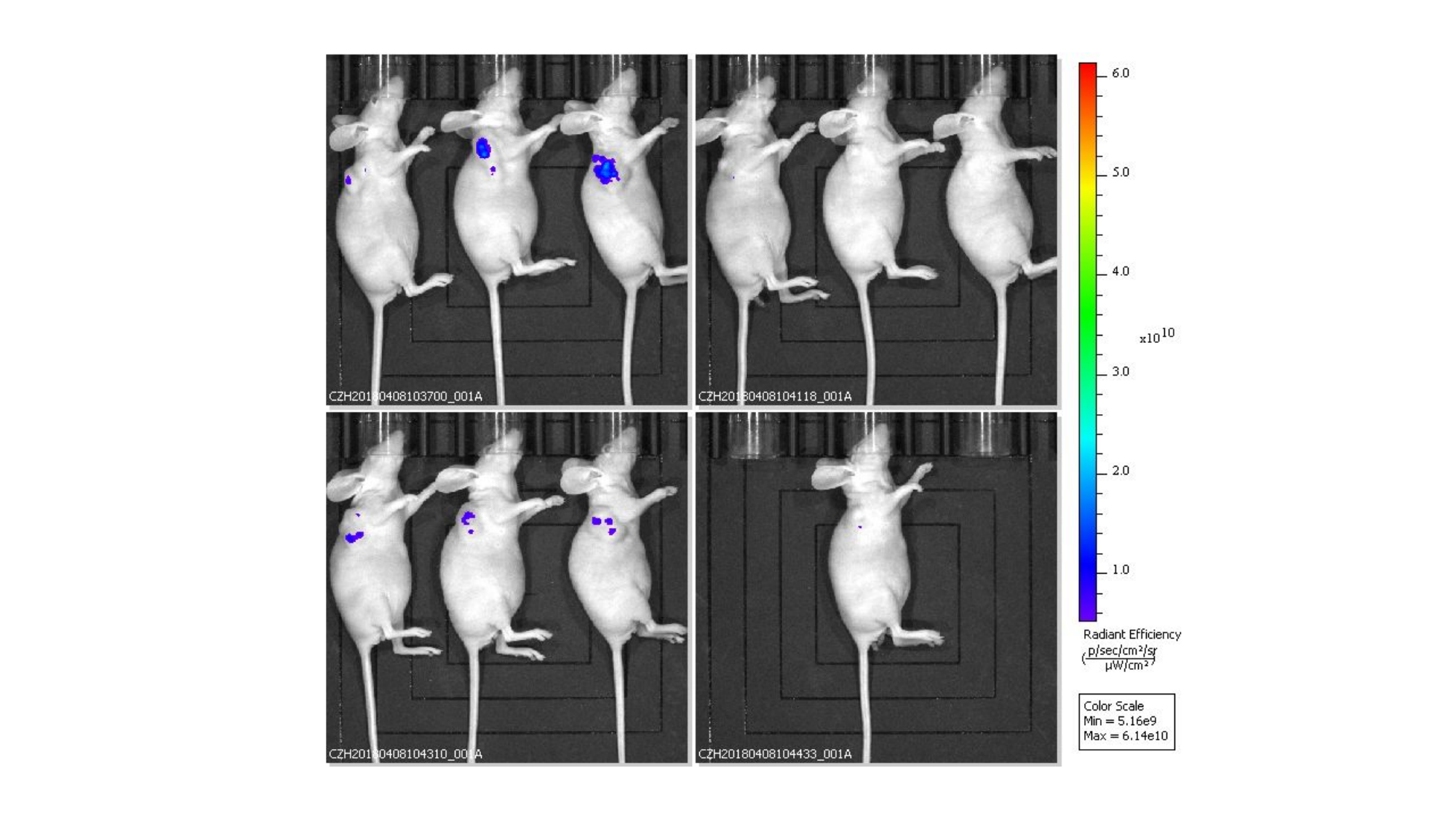

## Slide 6
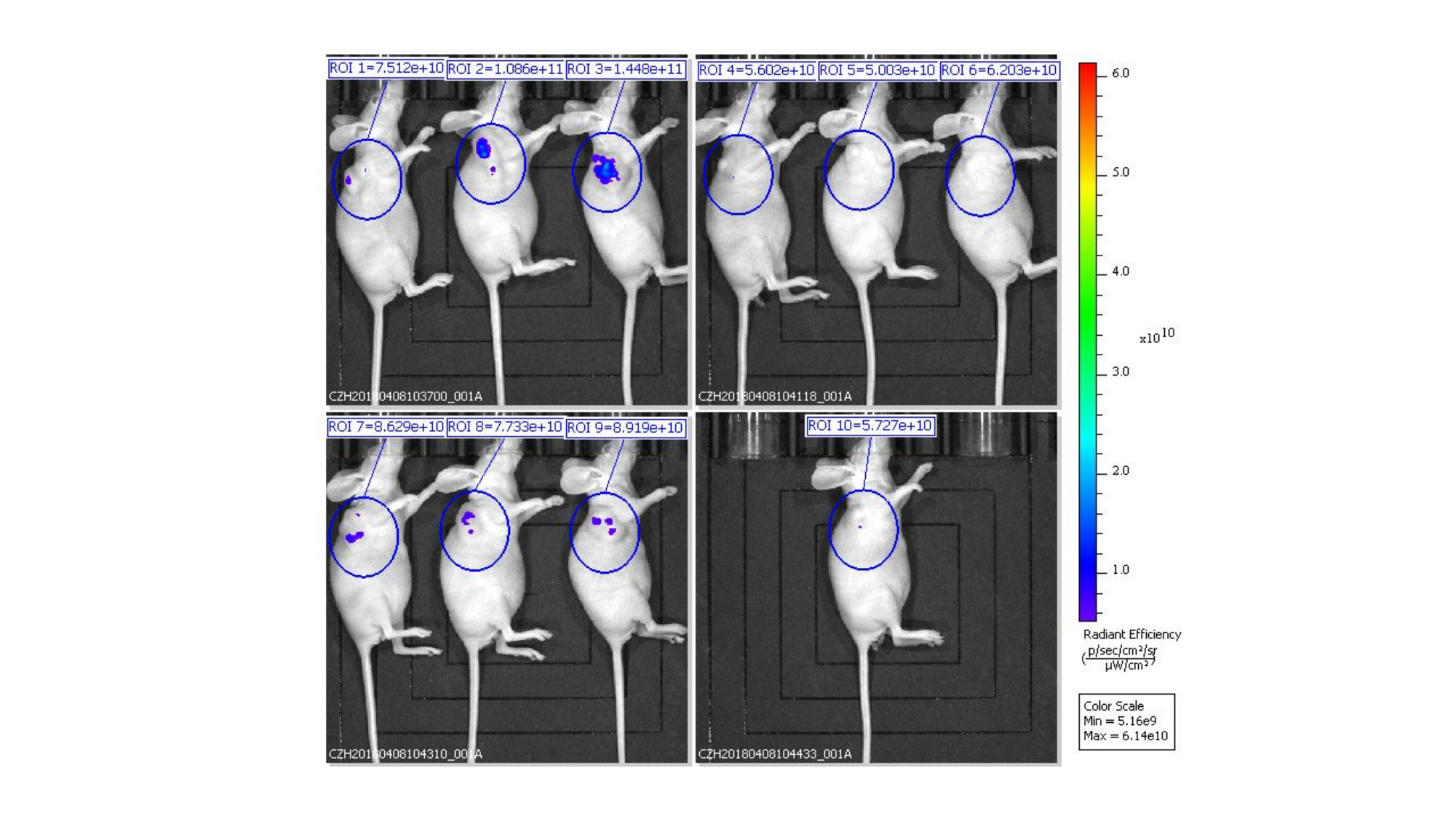

## Slide 7
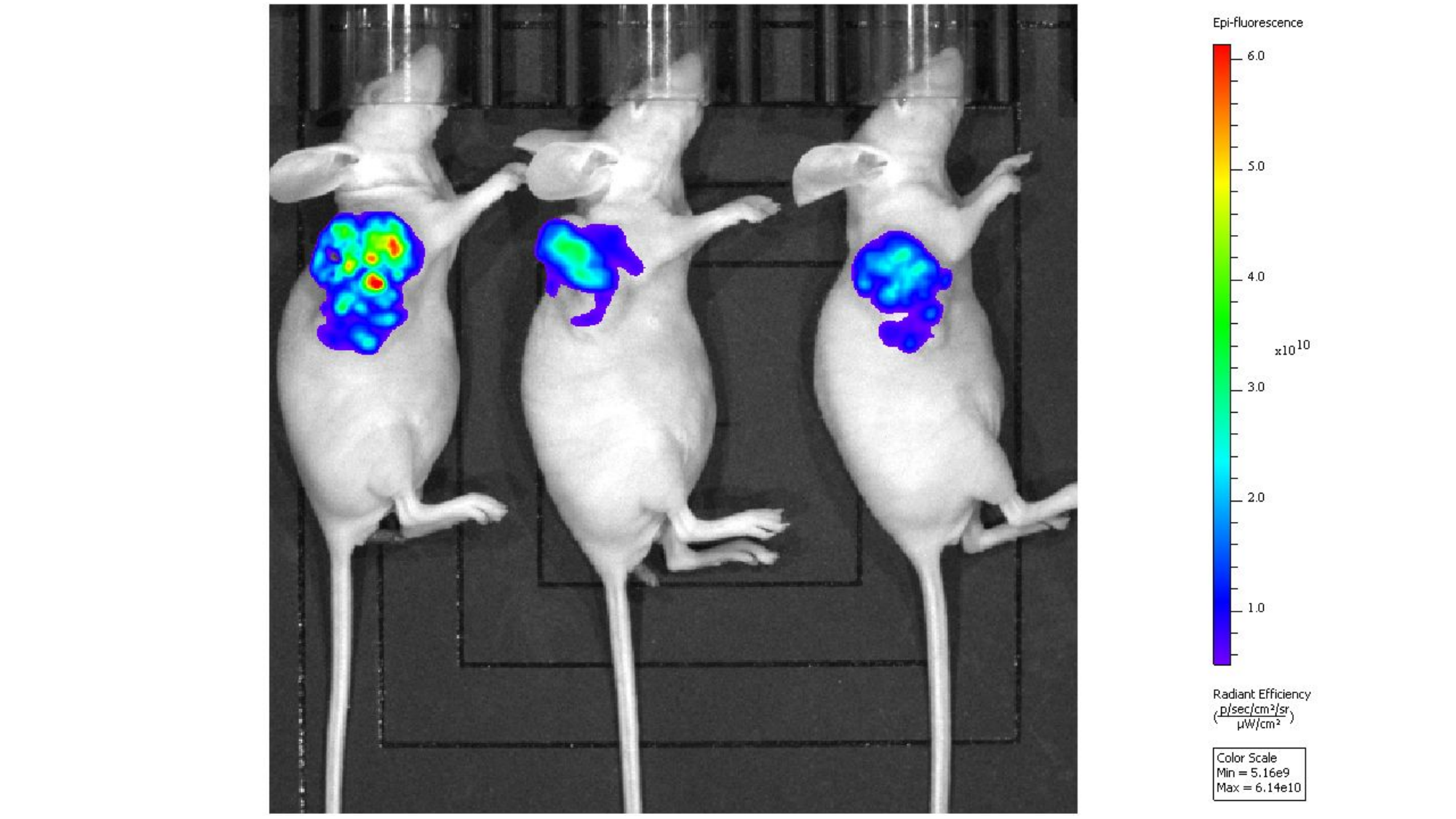

## Slide 8
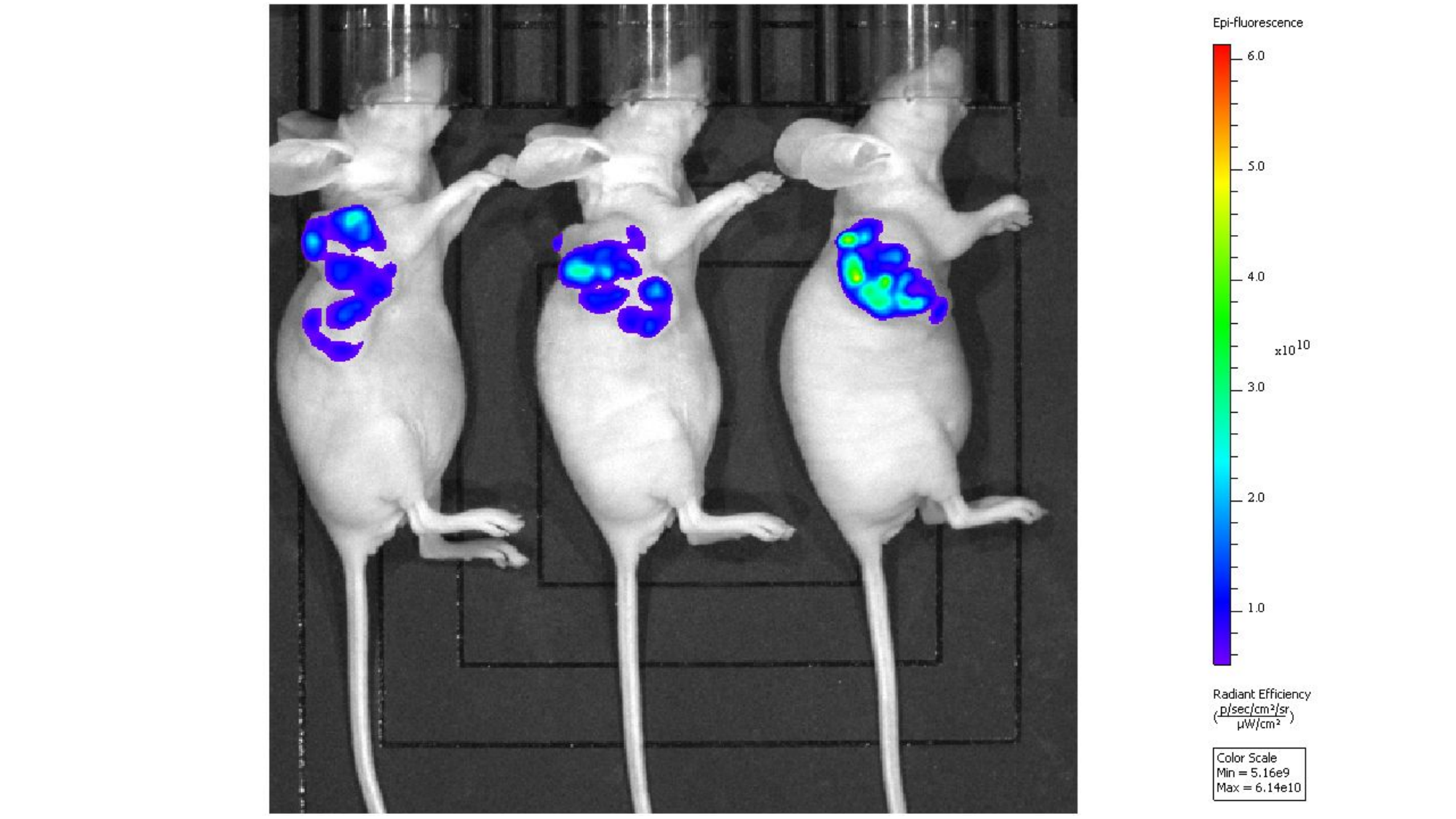

## Slide 9
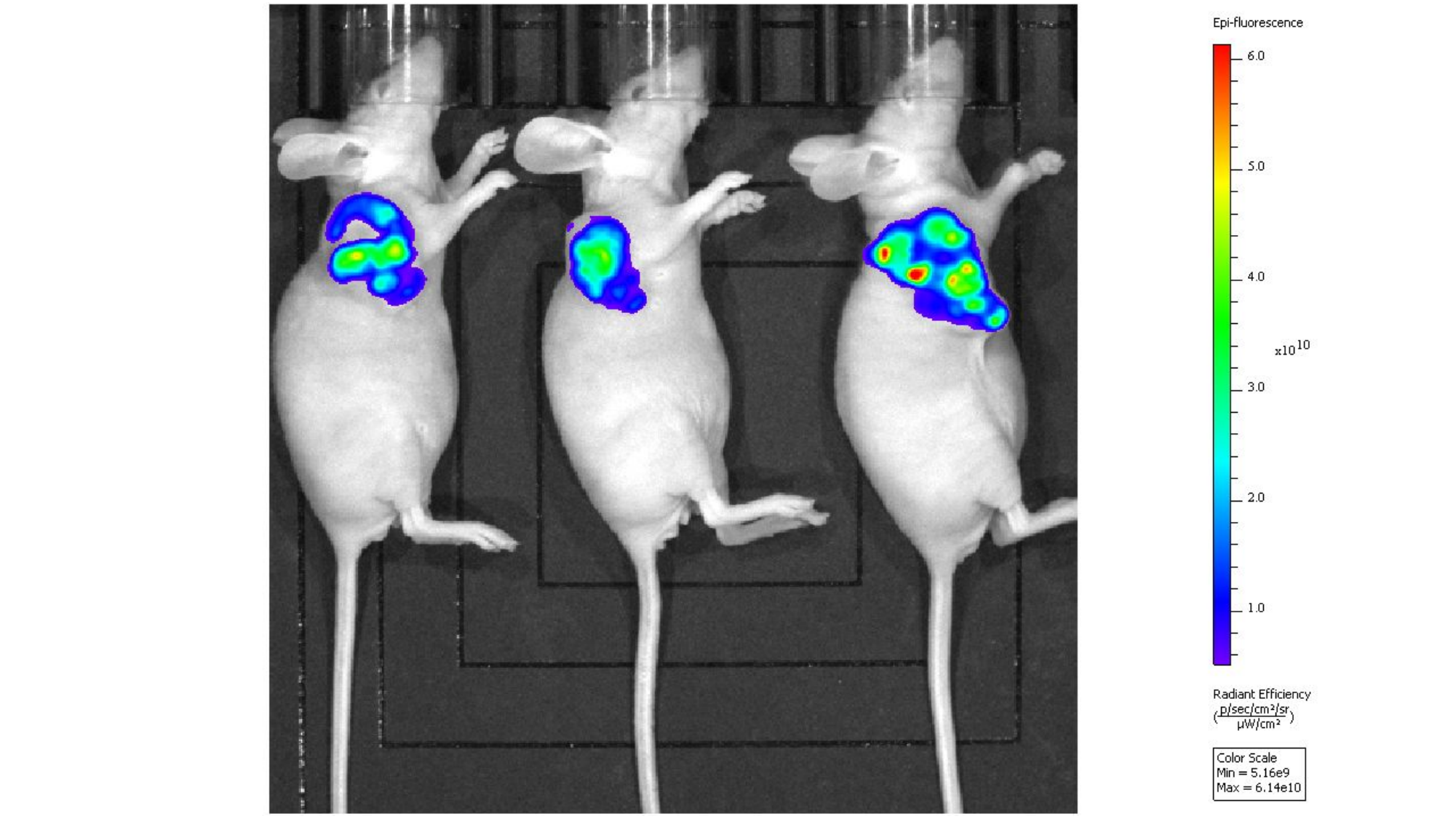

## Slide 10
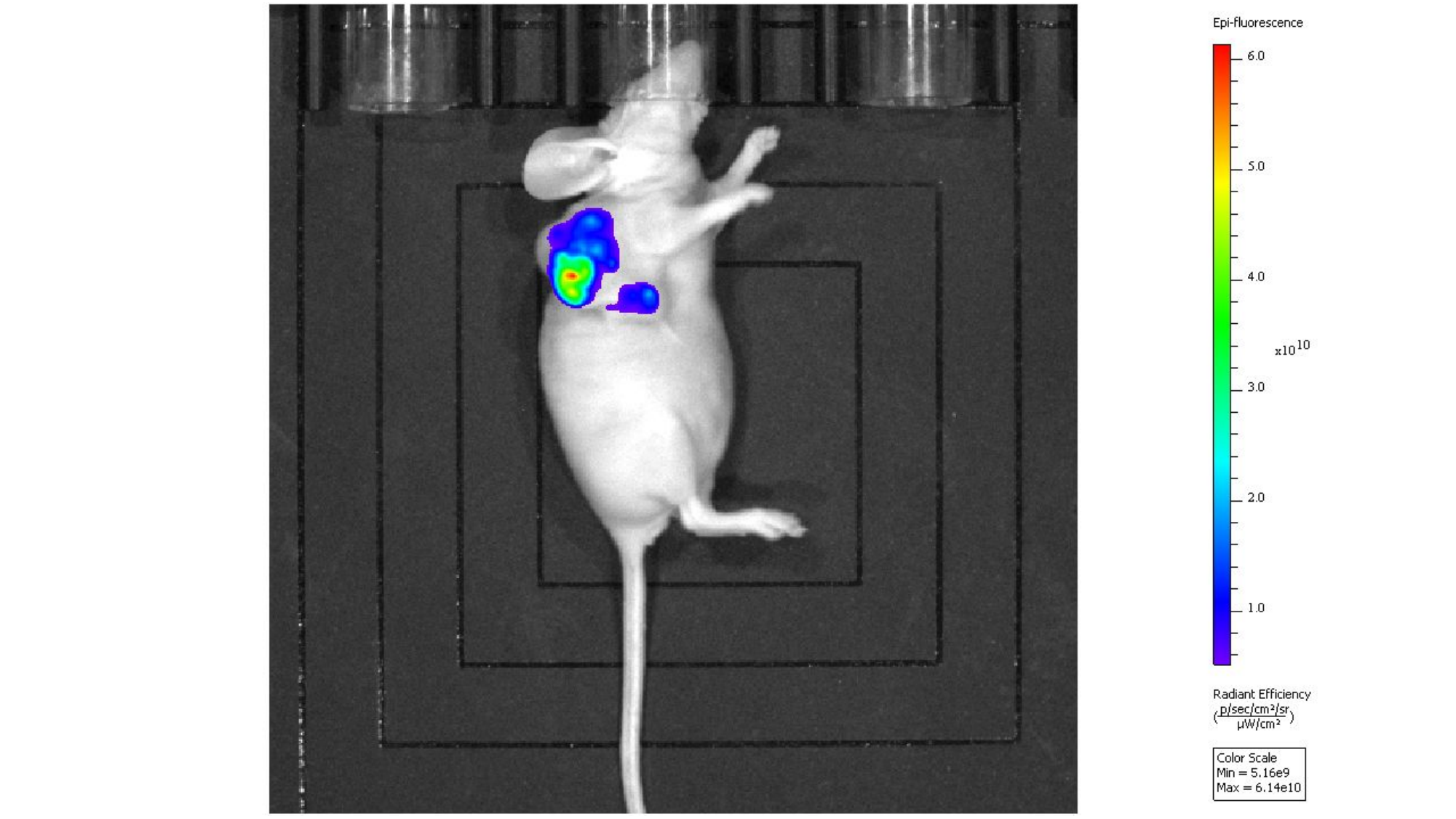

## Slide 11
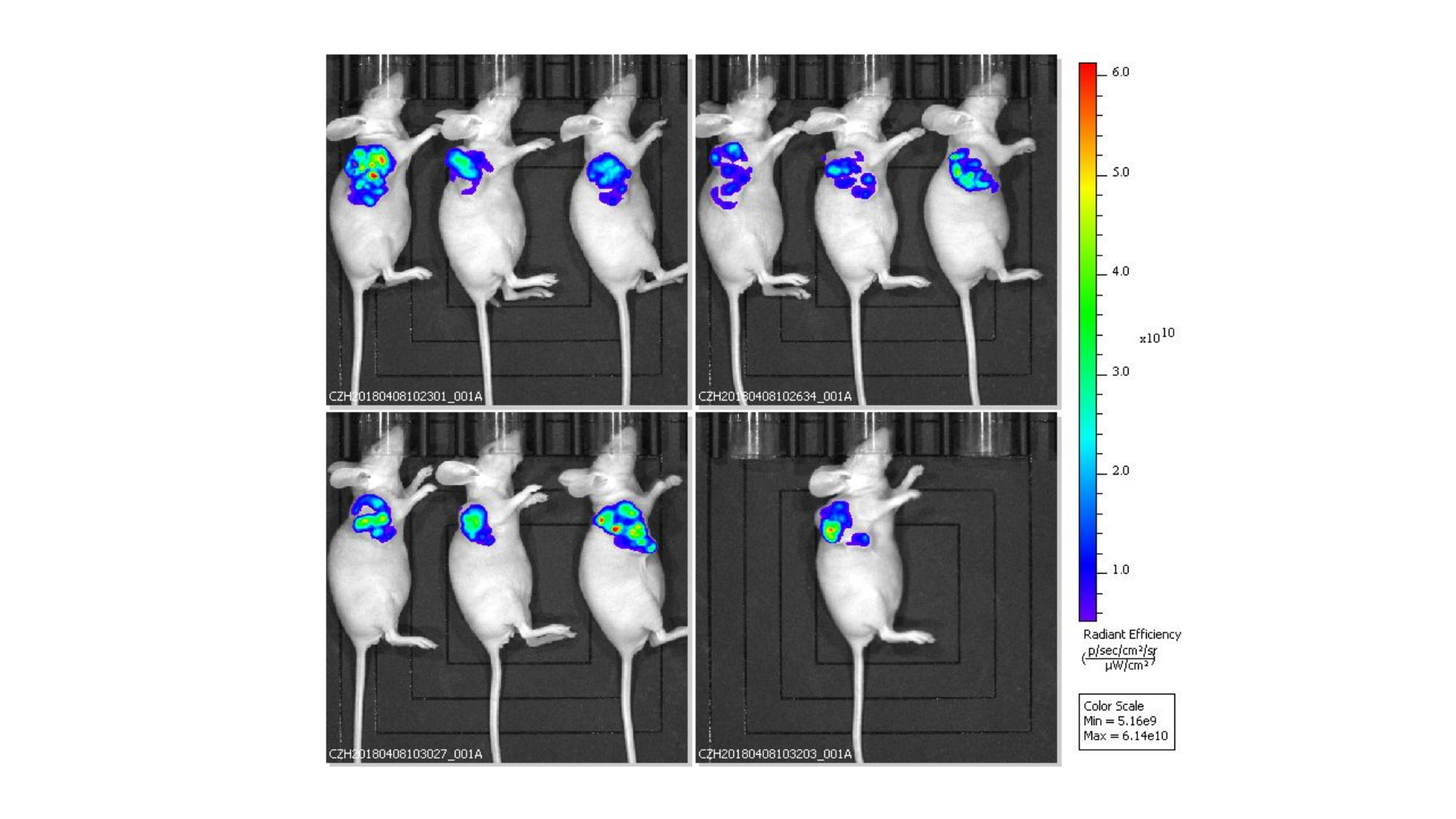

## Slide 12
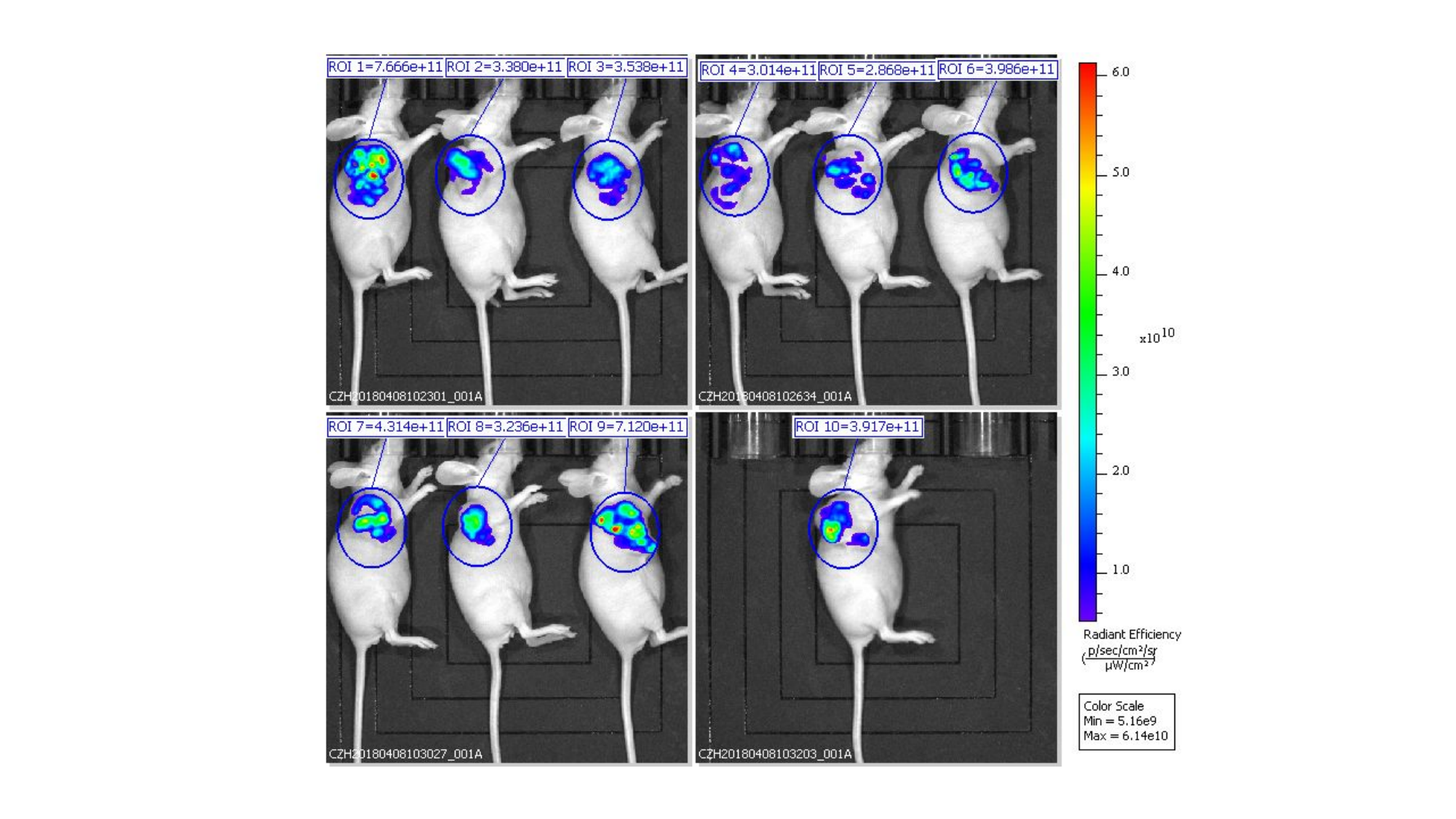

Supplement: Supplementary file 1 [file Presentation_1.zip › Fluorescence images of each group in nude mice subcutaneous tumor.PPTX]
